# Genome-wide identification and analysis of paclobutrazol-resistance gene family in cotton and the positive role of *GhPRE3* in salt stress and drought stress resistance

**DOI:** 10.1007/s10142-025-01701-2

**Published:** 2025-09-29

**Authors:** Shuaikang Geng, Jingjing Zhai, Bingkai Cui, Huiyun Shan, Zili Liu, Jiahui Li, Cong Wang, Yuling Liu, Pengtao Li, Quanwei Lu, Zihan Xiao, Xiaoping Pan, Renhai Peng, Yangyang Wei, Shoulin Hu

**Affiliations:** 1 College of Agriculture, Tarim University, Alar, 843300 China; 2 Anyang Institute of Technology, Anyang, 455000 China; 3 College of Life Sciences, Zhengzhou University, Zhengzhou, 450001 China; 4 Department of Biology, East Carolina University, Greenville, NC 27858 USA

**Keywords:** Paclobutrazol-resistance, Salt stress, Cotton, *GhPRE3*, VIGS

## Abstract

Compared with other transcription factors, much less studies have been performed on paclobutrazol-resistance (PRE), a subgroup of the extensive bHLH transcription factor gene family, and the research in cotton was also limited. By utilizing the PRE genes and their conserved domains identified in Arabidopsis, a total of 23, 22, 11, and 12 PRE genes were identified from two major cultivated cotton species and their two ancestors, respectively. The cotton PRE gene family was categorized into three subgroups based on evolutionary tree analysis. Motif and intron analyses indicated that the PRE gene has remained highly conserved throughout evolution. Collinearity analysis indicated that gene duplication, particularly through fragment replication, has significantly contributed to the expansion of the cotton PRE family. An exploration of the conserved elements within the PRE gene family uncovered numerous elements associated with plant stress resistance. Additionally, cotton transcriptome and qRT-PCR analysis showed that PRE genes were associated with a variety of abiotic stresses, including salt, drought, and cold treatments. Subcellular localization experiments indicated that the *GhPRE3* gene is associated with membrane proteins. Finally, we selected the *GhPRE3* gene for a VIGS experiment, which revealed that under salt stress and drought stress conditions, the wilting of leaves in the *GhPRE3-*silenced plants was significantly more severe than that observed in the control group, with T-AOC levels notably lower and MDA levels significantly higher. Overexpression of *GhPRE3* enhanced seed germination and root development in transgenic *Arabidopsis thaliana* under salt stress and drought stresses. This suggests that *GhPRE3* plays a positive regulatory role in cotton tolerance to salt and drought stressed, providing a reference for molecular genetic breeding of cotton with salt and drought tolerance.

**Supplementary Information:**

The online version contains supplementary material available at 10.1007/s10142-025-01701-2.

## Introduction

The adverse environmental conditions, such as drought, salinity, extreme cold, and high temperatures, significantly influence plant growth and development (Prakash et al. 2023). When plants are exposed to conditions that exceed their maximum tolerance, vital physiological processes like respiration and photosynthesis can be disrupted, potentially leading to plant mortality (Bashir et al. 2019; Godwin and Farrona 2020; Guo et al. 2021; Hill et al. 2024; She et al. 2024; Zhao et al. 2020; Zhou et al. 2024). Consequently, over the extensive course of evolution, plants have developed a variety of mechanisms to resist these abiotic stresses, enabling them to adapt proactively to harmful environmental conditions. For instance, in situations of drought and water scarcity, plants respond by closing their stomata and decreasing the osmotic potential of their cytoplasm to better tolerate the challenges posed by drought and elevated salinity levels (Roychoudhury et al. 2013). In response to abiotic stress, plants can modulate the expression of numerous genes through signal transduction pathways that depend on calcium ions (Ca^2+^), as well as through phospholipid signaling and protein phosphorylation or dephosphorylation mechanisms (Israelsson et al. 2006; Yamaguchi-Shinozaki and Shinozaki 2006). Within this signaling framework, certain early-response genes, including transcription factors such as NAC, WRKY, bHLH, and DREB, play a critical role. The activation or regulation of late-response genes occurs under the influence of these early-response genes, leading to physiological and biochemical alterations that ultimately enhance the plant’s resilience (Zhu 2002).

The bHLH protein family, characterized by an alkaline region responsible for DNA binding, also includes a helix-loop-helix domain that facilitates protein-protein interactions (Jones 2004). Proteins containing the bHLH domain typically function as either homodimers or heterodimers to regulate target gene expression. These proteins are involved in various physiological processes and play diverse roles in hormone biosynthesis, metabolism, and signal transduction within plants (Bai et al. 2012; Leivar and Quail 2011; Zhao et al. 2008). The PRE gene encodes a distinct basic helix-loop-helix (bHLH) protein, which is part of the second-largest family of transcription factors, known as bHLH (Atchley and Fitch 1997; Jones 2004; Ledent and Vervoort 2001). Within the Arabidopsis PRE family, six members have been identified: PRE1, PRE2, PRE3, PRE4, PRE5, and PRE6, each serving a specific function. However, during plant growth and development, these proteins exhibit functional redundancy. For example, PRE1, PRE2, and PRE4 act as target genes for the AP3/P1 complex in floral tissues and are subject to negative regulation, making them essential for proper flower development. In plants lacking the PRE4 gene, there is a significant reduction in chlorophyll levels, resulting in pale sepals and carpels (Hyun and Lee 2006; Lee et al. 2006; Mara et al. 2010; Ruzinova and Benezra 2003; Schlereth et al. 2010; Zhang et al. 2009). Despite this, the role of the transcription factor PRE remains largely unexplored in plant abiotic stress tolerance.

Cotton, recognized as a primary fiber source, is a significant cash crop. One strategy for enhancing the yield and quality of cotton is through gene function mining. However, there are less research on PRE gene family; as more and more genome sequencing and assembly, particularly as pan-genome development (Hu et al. 2024; Peng et al. 2021), it provides an extensive resource for genome-wide identification and function analysis of different gene families. In this research, based on the conserved domains of bHLH family genes and the PRE protein from *Arabidopsis thaliana*, we identified and screened PRE genes from four cotton species. First, we conducted protein characteristic analysis and compositional structure analysis of the screened PRE genes. Then, we performed evolutionary relationship analysis and grouping of the PRE gene family. Finally, through downloading cis-regulatory element and transcriptome database data, we analyzed the potential roles of PRE genes in abiotic stress. Additionally, we selected a representative gene *GhPRE3* for further functional verification, in which it was confirmed that the *GhPRE3* gene plays a positive role in cotton’s salt and drought resistance. This work serves as a reference for advancing the functionality of the transcription factor PRE and contributes to enriching the data available in the cotton gene bank. This will serve as a reference gene for the genetic improvement project aimed at enhancing cotton tolerance to different abiotic stresses, including drought and salinity.

## Materials and methods

### Identification and physicochemical properties of PRE subfamily genes in cotton

The genome sequences for *Gossypium barbadense* (H7124_ZJU), *G. hirsutum* (TM-1_WHU), *G. arboreum* (A2_WHU), and *G. raimondii* (D5_HAU) were obtained from CottonMD (https://yanglab.hzau.edu.cn/CottonMD) (Yang et al. 2023). The gene sequences for *Arabidopsis* AtPRE were extracted from the Plant Transcription Factor Database (PlantTFDB; http://planttfdb.gao-lab.org) (Tian et al. 2020). Utilizing the specific domain associated with PRE family genes, namely the HLH domain (PF00010), the Hidden Markov Model (HMM) profile for PF00010 was sourced from InterPro (https://www.ebi.ac.uk/interpro/entry/pfam) (Paysan-Lafosse et al. 2023). The coding domain sequences (CDS) containing PF00010 across the four cotton species were analyzed using the HMM search feature in the TBtools software (ToolKit Biologists Tools) (Chen et al. 2023) to identify potential PRE family members. For the identification of conserved domains, the amino acid sequences were analyzed with CDD-Search available in NCBI and TBtools software, and the classification of PRE family members was based on their conserved domain types and counts. The physiological and biochemical parameters of all cotton PRE gene proteins, including molecular weight, isoelectric point, instability coefficient, fat coefficient, and average overall hydrophilicity, were evaluated using the online tool Expasy-ProtParam (https://web.expasy.org/protparam/) (Wilkins et al. 1999). Subcellular localization predictions for cotton PRE gene proteins were conducted using the online resource WOLF PSORT (https://wolfpsort.hgc.jp/), and the results regarding protein physiology, biochemistry, and localization predictions were compiled in an Excel file.

### Construction of phylogenetic tree of cotton PRE family genes

Phylogenetic trees representing the PRE subfamily genes from four cotton species were constructed using MEGA11 software (Tamura et al. 2021). For the multiple sequence alignment of protein-coding regions of PRE genes across the four cotton species (*G. barbadense*, *G. hirsutum*, *G. arboreum*, and *G. raimondii*), the ClustalW tool was utilized with default settings. The evolutionary relationships were inferred using the Neighbor-Joining method. The percentage of replicate trees indicating the clustering of associated taxa in the bootstrap analysis (with 1000 replicates) is displayed alongside the branches. Evolutionary distances were calculated using the JTT matrix-based approach, expressed as the number of amino acid substitutions per site. Ambiguous positions were excluded from each sequence pair through the pairwise deletion option. The resulting evolutionary tree was further enhanced and visualized using the online Evolview tool (https://www.evolgenius.info/evolview-v2/#mytrees//) (He et al. 2016).

### The physical distribution of cotton PRE family genes on the chromosome

The Gene Location feature of TBtools was employed to collect location data for PRE family genes from four cotton species, utilizing the gene annotation files associated with these. Furthermore, chromosomal positioning and visualization were accomplished with a single click.

### Analysis of conserved motifs and gene structures of PRE family genes

Based on the previously extracted location data of family members, TBtools was employed to analyze the exon-intron architecture of each PRE gene. To identify conserved motifs within the PRE protein sequence, the online platform MEME (https://meme-suite.org/meme/tools/meme) was utilized (Bailey et al. 2015). The parameters were set to a maximum base ordinal number of 10, a minimum base sequence length of 6, and a maximum length of 50, while the remaining settings were maintained at their defaults. The output file was downloaded in XML format, facilitating the visualization of the conserved motifs and gene structure using TBtools. The pafm domain analysis of the entire family members of PRE was conducted through the online website InterPro.

### Collinear analysis of PRE gene in cotton

The chromosome framework was acquired using TBtools-fasta stats, while the gene association file was generated through TBtools-MCScanX, and ultimately, the visualization was performed with the TBtools-Advanced Circos tool.

### Analysis of promoter Cis acting elements of PRE family genes

Based on the location details of the PRE genes, the upstream 2000 bp fragment was extracted from the genome sequence file using TBtools. Furthermore, the online tool PlantCARE (http://bioinformatics.psb.ugent.be/webtools/plantcare/html/) (Lescot et al. 2002) was employed to predict the cis-acting elements within the promoter region of the genes, and the predicted regulatory elements were subsequently visualized using TBtools.

### Analysis of abiotic stress expression of PRE genes in G. hirsutum

The GhPRE gene ID from the screened *G. hirsutum* was submitted to the CottonMD database. Expression data for *G. hirsutum* TM-1 under treatments of heat (37 °C), chilling (4 °C), sanility (NaCl), and drought (PEG), from NCBI BioProject ID: PRJNA490626, were downloaded and the transcriptome data was standardized (Table S1). The expression level of each gene was represented using log2 (TPM + 1). Heat maps were generated using TBtools software.

### Plant materials and growth conditions

Both *G. hirsutum* L. acc. TM-1, and the tobacco (*Nicotiana benthamiana* were are provided by the Genetics Research group of Anyang Institute of Technology. TM-1 was used for qRT-PCR and VIGS experiments, and *Nicotiana benthamiana* was used for subcellular localization experiments. The seeds of TM-1 were immersed in water for 48 h, after which they were planted in pots filled with soil. The seedlings were cultivated at a temperature of 28℃, with a photoperiod of 16 h of light and 8 h of darkness, until two true leaves fully expanded. Following this period of consistent growth, the plants were separately treated with 350 mM NaCl, 20% PEG6000, and 4℃, and incubated at 28℃. Cotton leaves were harvested at various time points (0 h, 1 h, 3 h, 6 h, 12 h, 24 h and 48 h) using liquid nitrogen for preservation before being stored at −80℃ in a freezer. Seeds of *Nicotiana benthamiana* were sown in nutrient-rich soil at 21℃, also receiving 16 h of light and 8 h of darkness. Once the *Nicotiana benthamiana* seedlings developed two leaves, they were transferred into individual nutrient bowls, with one plant allocated per bowl. These seedlings were irrigated with a nutrient solution enriched with phosphate fertilizer to facilitate the growth of sufficiently large leaves. Subcellular localization studies were conducted when the leaf area reached about 4 cm².

### RNA extraction and quantitative reverse transcription polymerase chain reaction (qRT-PCR)

Total RNAs were isolated from cotton leaves using the FastPure Universal Plant Total RNA Isolation Kit (Vazyme, Nanjing, China). Approximately 1–2 µg of total RNAs were converted into cDNAs through reverse transcription with the Uni All-in-One First-Strand cDNA Synthesis SuperMIX for qPCR (Transgen, Beijing, China), following the manufacturer’s guidelines. The resulting cDNAs served as templates in qPCR reactions that employed gene-specific primers, with *GhActin7* serving as a reference gene, whose stability was tested and confirmed by an online computational program RefFinder (Xie et al. 2023). Each qRT-PCR reaction was conducted in triplicate, and the gene-specific primers used for this analysis are detailed in Supplemental Data Table S2. The real-time fluorescence quantitative data were analyzed using the -△△Ct method, and the results were organized alongside those from the cotton database using an Excel file.

### Subcellular localization of GhPRE3

The plasmid vector used for the subcellular localization of the genes was pCAMBIA2300-DsRed2, which was modified in our laboratory. The complete *GhPRE3* gene was employed as the product sequence, with its termination codon omitted. Xba I and BamH I were utilized as primers for designing the enzyme restriction sites of the plasmid (Table S2). The *GhPRE3* gene was then linked to the pCAMBIA2300-DsRed2 vector using the ClonExpress^®^ II one-step cloning kit. Following this, the recombinant plasmid was introduced into DH5α competent cells. Confirmation of the recombinant results was achieved through PCR amplification and sequencing. Positive clones were selected, and the plasmids they contained were extracted and transformed into the *Agrobacterium* strain GV3101. *Nicotiana benthamiana* leaves were subsequently infected using 1 ml syringes. Two days after the infection of the *Nicotiana benthamiana* leaves, 1 cm² sections of the infected leaves were harvested, and the red fluorescent signal emitted by the DsRed2 fusion protein was observed using a Leica DMi8 confocal laser scanning microscope (Leica, Wetzlar, Germany).

### Using virus-induced gene silencing (VIGS) to test GhPRE3 gene function

A fragment of 249 bp from the *GhPRE3* gene was inserted into the TRV2 vector to create the TRV2: *GhPRE3* construct, utilizing the Kpn I and Xba I restriction sites. Primers were designed using SnapGene software (Table S2) (Kumagai et al. 1995; Tian et al. 2024). Similarly, the TRV2: PDS construct was assembled as a visual marker to assess silencing efficiency (Pang et al. 2013). The negative control used was TRV2: 00, which serves as an empty vector. All vectors were introduced into GV3101. We injected the cotyledons of 7-day-old TM-1 seedlings at a temperature of 28 °C, followed by a 24-hour treatment at 24 °C in the dark. Cotton plants that received the TRV2: PDS injection displayed an albino phenotype two weeks post-infiltration. Upon reaching the three-leaf stage, WT, TRV2:00 and TRV2:*GhPRE3* strains were treated with 350 mM NaCl and 20% PEG6000 solution until the phenotype was obvious, and the leaf samples were photographed and obtained.

### Construction of GhPRE3 Overexpression Vector and Transformation in Arabidopsis thaliana

*Arabidopsis thaliana* ecotype Columbia (Col-0) plants were obtained from our laboratory stock. All the plants were grown in the growth chamber under controlled conditions at 22 °C day/19°C night with 16 h of light per 24 h and 50% humidity. The target gene fragment of *GhPRE3* from the cDNA of TM-1 was amplified using GhPRE3-2300 primers (Table S2) and constructed it into the pCAMBIA2300 overexpression vector. After sequencing the plasmid to confirm that the clone is in frame and oriented correctly, the recombinant construct was transformed into *Agrobacterium tumefaciens* strain (pGV3101) via chemical transformation. The floral dip method was used to generate overexpression transgenic plants and select the transformed resistant plants using kanamycin plates (Clough and Bent 1998). Positive transgenic lines were selected when the resistant: sensitive segregation ratio was 3:1.

To evaluate the sensitivity of *Arabidopsis thaliana* overexpressing *GhPRE3* to salt and drought stresses, germination tests and root length growth assays were conducted. Salt treatment during the germination period was performed by adding NaCl to 0.5 MS medium to achieve final concentrations of 100 mM, 150 mM, and 200 mM, while drought treatments were conducted by adding mannitol to 0.5 MS medium to achieve final concentrations of 250 mM, 300 mM, and 350 mM. For germination rate determination, WT and three overexpressing *GhPRE3 Arabidopsis* seeds were sterilized and then placed in a low-temperature environment for 48 h. Afterward, they were evenly sown on 0.5 MS medium of different concentrations. Radicle emergence was used as the indicator of germinated seeds, and seedling germination rates were observed and calculated daily for 9 days. For root length growth testing, WT and three overexpressing seedlings were sterilized and cultured in darkness for two days, followed by two days of culture on vertical MS medium plates. Subsequently, more than three seeds with similar root lengths were transferred to MS medium containing different salt and mannitol concentrations for 22 days. Root lengths were measured using a ruler. The entire experiment was repeated three times. One-way ANOVA was used to analyze statistical significance between samples, and post-hoc tests were conducted (Kong and Ramonell 2022; Qu et al. 2020, 2023).

## Results

### Identification and physicochemical properties of PRE subfamily genes in cotton

Based on six AtPRE protein sequences and using the bHLH domain (PF00010) as a reference, a total of 23, 22, 11, and 12 PRE genes were identified in *Gossypium hirsutum* (TM-1), *G. barbadense* (H7124), *G. arboreum* (A2), and *G. raimondii* (D5), respectively. The gene IDs were assigned to the identified genes as *GhPRE1* through *GhPRE23* for *G. hirsutum*, *GbPRE1* through *GbPRE22* for *G. barbadense*, *GaPRE1* through *GaPRE11* for *G. arboreum*, and *GrPRE1* through *GrPRE12* for *G. raimondii*, following a sequential numbering system. An analysis of the physical and chemical characteristics indicated that the amino acid count ranged from 87 to 101, with predicted molecular weights between 9678.90 and 11442.55. Additionally, the isoelectric points varied from 5.18 to 10.35, while the instability coefficients ranged from 53.36 to 91.75 (with values exceeding 40 suggesting potential instability), indicating instability across all identified PRE genes (Table S3).

### Phylogenetic analysis of PRE subfamily genes in cotton

**Fig. 1:**
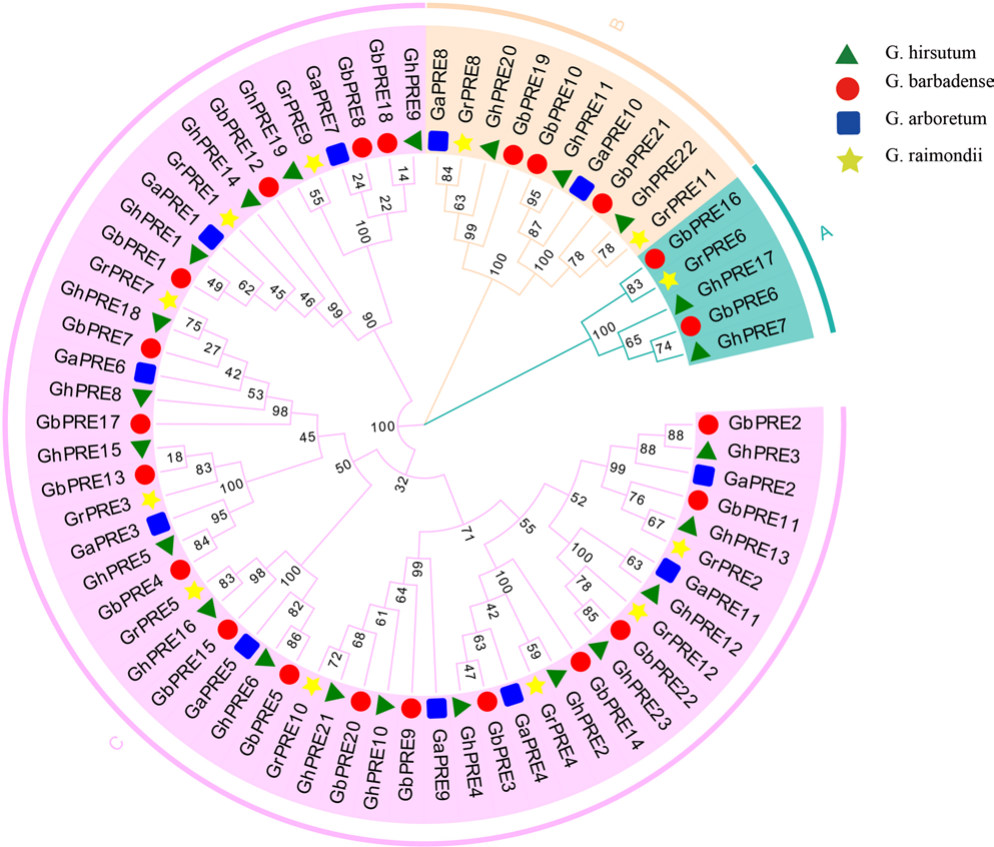
Phylogenetic tree of PRE genes in 4 cotton species

To investigate the evolutionary relationship of the PRE genes in cotton, a phylogenetic tree was constructed. The analysis revealed that the PRE genes from the four cotton species could be classified into three distinct subgroups (Groups A-C; Fig. 1). Notably, the C subgroup contained the highest number of PRE genes (53), while the A subgroup had the fewest (5), the B subgroups contained 10 PRE genes. Additionally, the number of tetraploid cotton genes in each subgroup is approximately double that of the diploid cotton PRE genes, highlighting the evolutionary connection between tetraploid and diploid cotton. It was also observed that the PRE genes from *G. hirsutum* and *G. barbadense* consistently clustered together.

### Phylogenetic relationships, conserved motif composition, and gene structure analysis of PRE genes

**Fig. 2:**
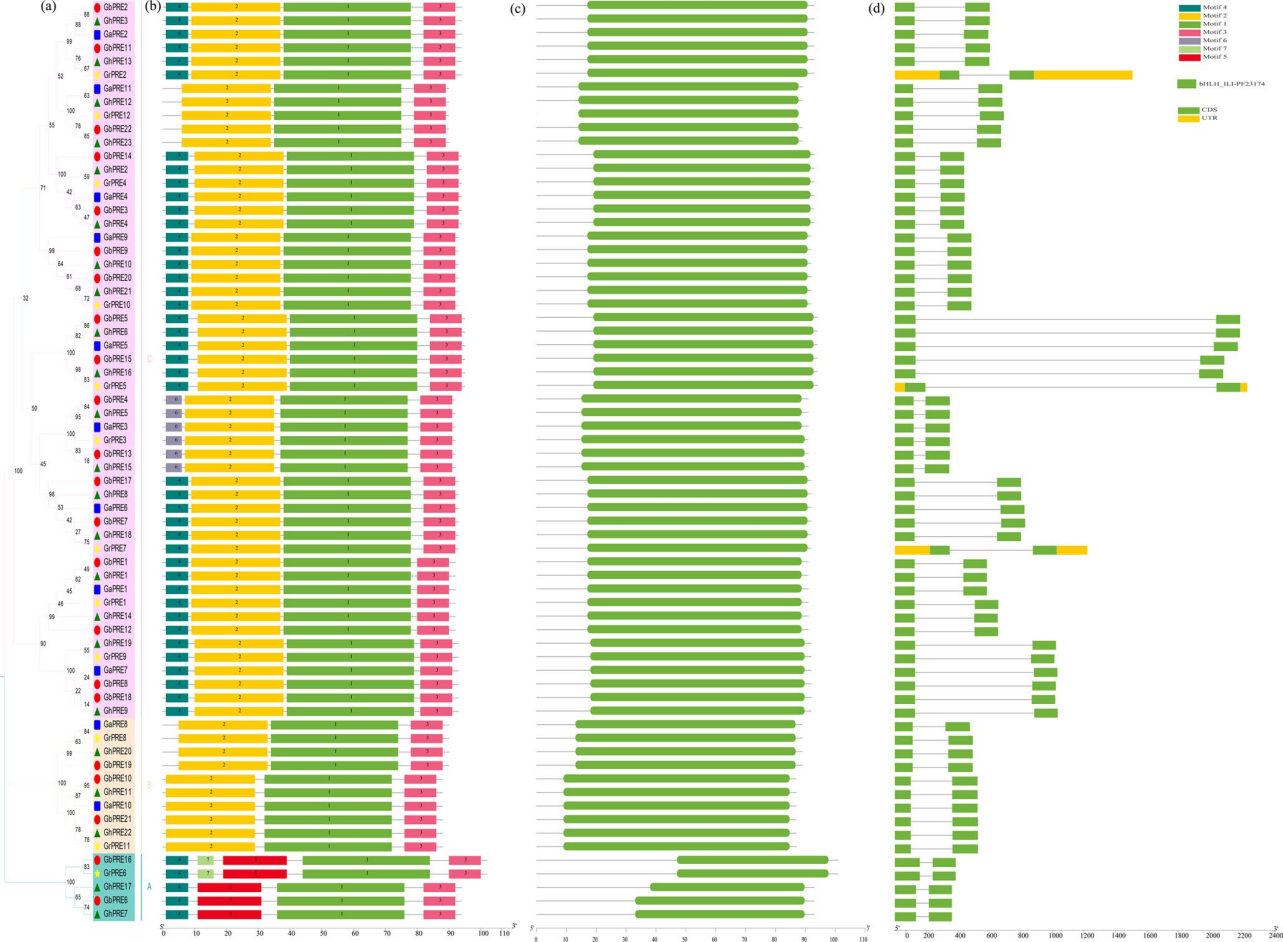
Structural triple diagram of PRE family in cotton. (**a**) Phylogenetic relationship of proteins encoded by PRE in cotton. (**b**) Conserved motif of PRE in cotton. (**c**) Pafm domain. (**d**) Conserved domains of proteins encoded by PRE in cotton

To gain a deeper understanding of the evolution and structural variability of the PRE gene subfamily, we conducted an analysis of the MOTIF composition and exon/intron structures in *G. hirsutum*, *G. barbadense*, *G. arboreum*, and *G. raimondii*. The conservative motif evaluation identified a total of seven distinct motifs, labeled from motif 1 to motif 7. At the same time, MOTIF and structure were classified and mapped according to gene evolutionary tree relationship (Fig. 2a) The results indicated that both motif 1 and motif 3 were present in all analyzed genes, whereas motif 7 was unique to the *GbPRE16* and *GrPRE6* genes, suggesting a notable degree of conservation in the structure of the PRE genes (Fig. 2b). The analysis of the PAFM conserved domain revealed that motifs 1 and 3 are the essential components of the HLH domain (Fig. 2c). An examination of introns and exons revealed that only three of the genes contained introns, while the remaining genes were composed solely of exons (Fig. 2d). Overall, the analysis of motifs and introns determined that the PRE genes has remained largely conserved throughout its evolutionary history.

### Chromosomal mapping of PRE subfamily genes in cotton

**Fig. 3:**
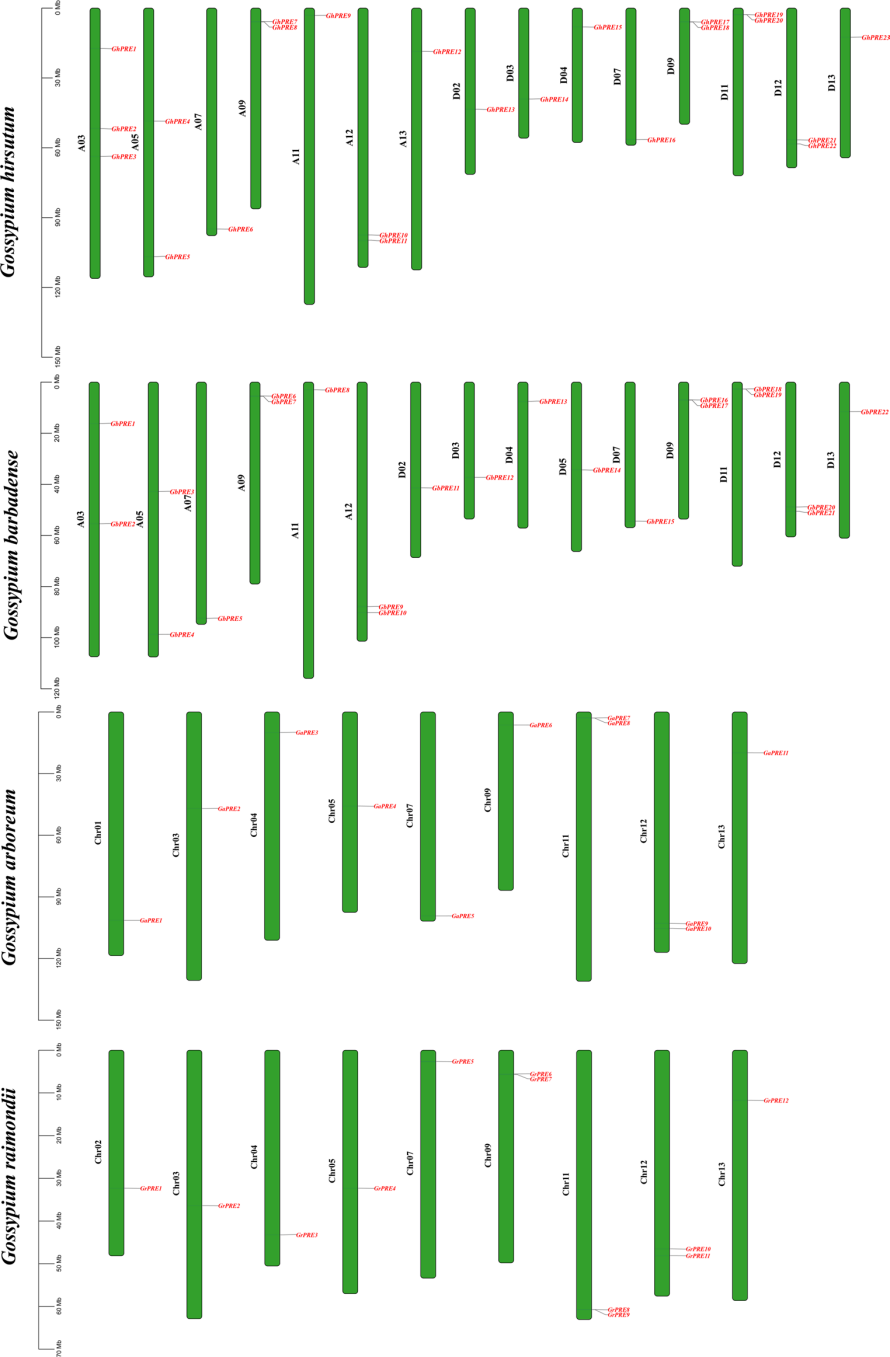
Distribution of PRE gene on cotton chromosome. The left vertical bar indicates the size of the chromosomes in Mb, with the chromosome number on the left side of each chromosome. The corresponding cotton is on the left side of the picture

To gain a clearer understanding of the distribution of the PRE genes across each chromosome, we developed a gene chromosome map. Our analysis revealed that only one or two PRE genes were present on each chromosome (Fig. 3). In *G. hirsutum*, a total of 23 PRE genes were unevenly distributed across 15 chromosomes, with 12 genes located on 7 chromosomes within the At sub-genome and 11 genes on 8 chromosomes in the Dt sub-genome. *G. barbadense* contains 22 genes distributed across 15 chromosomes, comprising 10 genes found on 6 chromosomes of the At sub-genome and 12 genes on 9 chromosomes of the Dt sub-genome. Additionally, *G. arboreum* exhibits 11 genes scattered across 9 chromosomes, while *G. raimondii* has 12 genes unevenly dispersed over 9 chromosomes.

### Collinear analysis of PRE gene in cotton

**Fig. 4:**
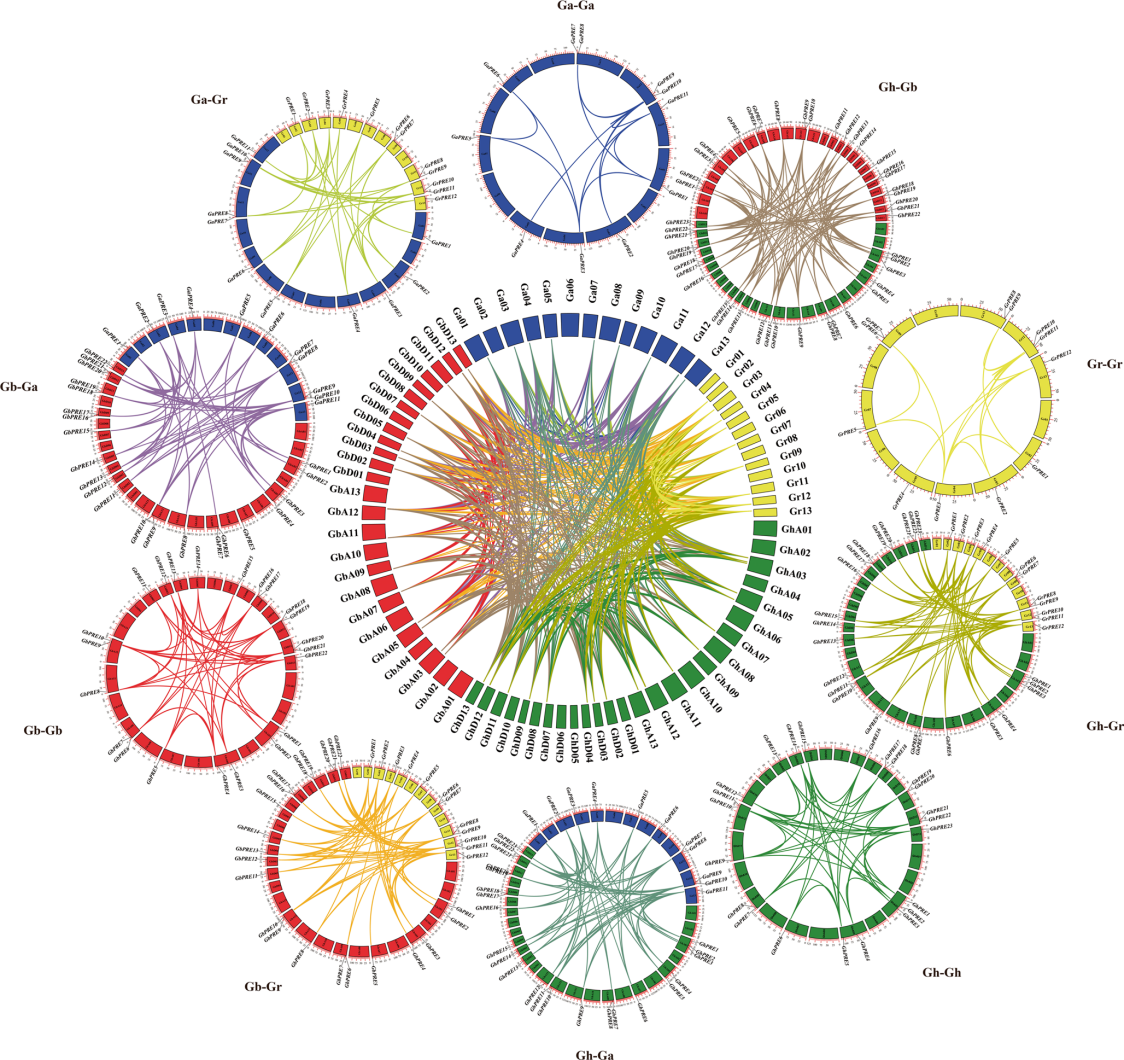
The analysis focused on the collinearity of repetitive gene pairs within the PRE gene across four species of cotton. Chromosomes from various cotton species were assigned distinct colors, and lines of different hues illustrated the divergent relationships existing both within and between the cotton species

Fragment replication, tandem replication, and whole genome replication are the primary mechanisms driving the growth of gene families. In tetraploid cotton, the number of PRE genes is double that observed in diploid cotton, indicating an expansion of the PRE gene family during the process of polyploidization in cotton, as the same as other transcription factor gene family (Chen et al. 2024; Mehari et al. 2023; Sun et al. 2023, 2025). To investigate this expansion, TBtools was employed to identify duplicate gene pairs, followed by an intra-species collinearity analysis of PRE genes across four cotton species. The analysis of intraspecies duplicate genes revealed that upland cotton possesses 50 duplicate genes (Fig. 4), while Island cotton has 48 pairs, Asian cotton has 16 pairs, and Raymond’s cotton exhibits 17 pairs. The interspecific gene analysis indicated 99 pairs of repeated genes between upland and Island cotton, 68 pairs between upland and Asian cotton, 80 pairs between upland and Raymond cotton, 64 pairs among Island, Asian, and Raymond cottons, 78 pairs between Island and Raymond cotton, and 39 pairs between Asian cotton and Raymond cotton. Overall, 559 pairs of repeated genes were identified across the four cotton species, with no tandem repeat genes detected. These findings underscore the significant role of gene duplication, particularly fragment replication, in the expansion of the cotton PRE gene family.

### Analysis of cis-acting elements of cotton PRE gene promoters

**Fig. 5:**
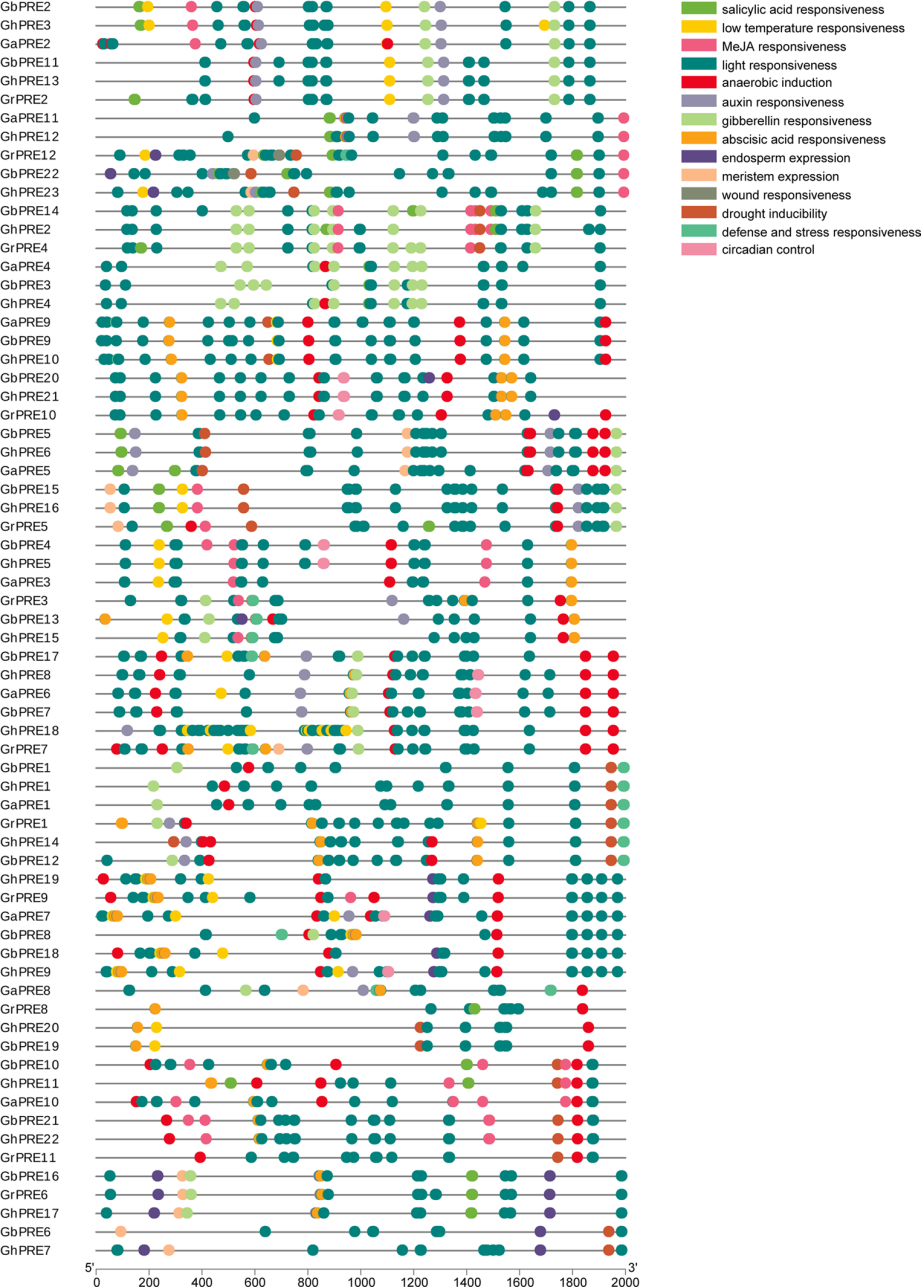
Cis elements in the promoter of cotton PRE Gene. Different colors represent different response elements

To investigate the role of the PRE genes, we utilized the PlantCARE website to conduct a predictive analysis of the 2000 bp upstream region of the coding area for the PRE family genes, thereby deriving the original regulatory cis-elements. Numerous cis-responsive elements were identified, which can generally be categorized into auxin response elements, erythromycin response elements, elements responsive to low temperatures, salicylic acid response elements, anaerobic response elements, jasmonic acid elements, light-responsive elements, abscisic acid response elements, as well as defense and stress-related elements relevant to plant growth and development, drought response, wound healing, physiological regulation, hypoxic-specific induction, and cell cycle induction (Fig. 5). Given the significant number of promoters associated with abiotic stress, we thought that these gene family maybe associated with abiotic stresses.

### Expression of PRE genes under stress treatments in TM-1 based on transcriptome database

**Fig. 6:**
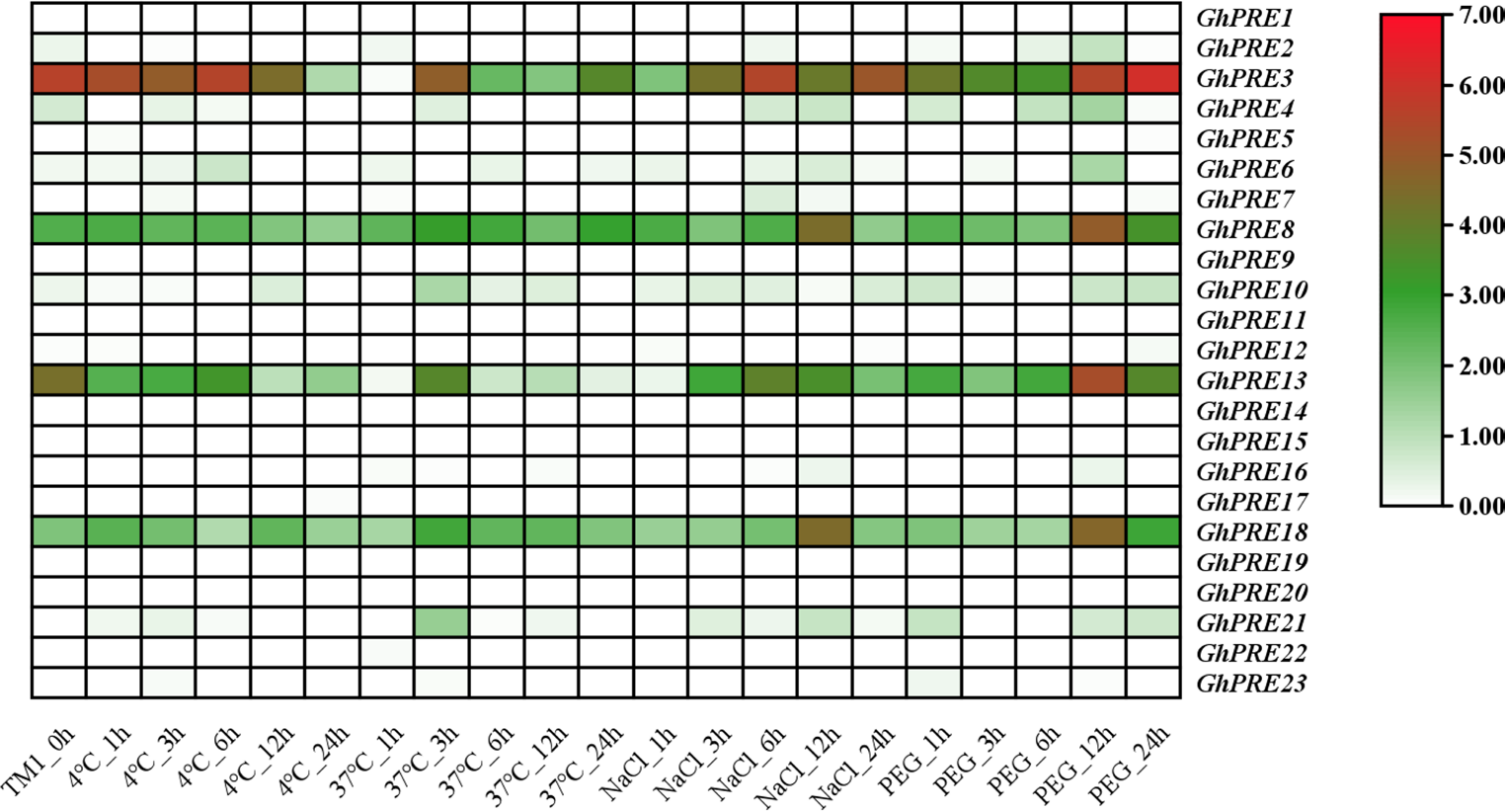
Analysis of abiotic stress expression of PRE gene in *G. hirsutum*

The expression data of the GhPRE genes in TM-1 subjected to abiotic stress were analyzed using the cotton database, and a heat map was generated with TBtools software. It was noted that there is limited knowledge regarding the majority of data associated with the PRE gene, underscoring the scarcity of research on the PRE genes in cotton at this time. The analysis of the PRE gene expression calorigram revealed a significant expression of *GhPRE3*, which exhibited a wavy pattern across all treatments (Fig. 6). During exposure to low temperatures at 4 °C and NaCl treatment, the expression initially declined, followed by an increase that peaked after 6 h, before ultimately decreasing. Under a treatment of 37 °C, the expression dropped sharply at the 1-hour mark, peaked at 3 h, and then began to decline. In the drought simulation using PEG treatment, the expression initially decreased firth and then increased after 12 h, and surpassed the baseline by the 24-hour mark.

### qRT-PCR analysis of PRE genes in TM-1 under different abiotic stresses

**Fig. 7:**
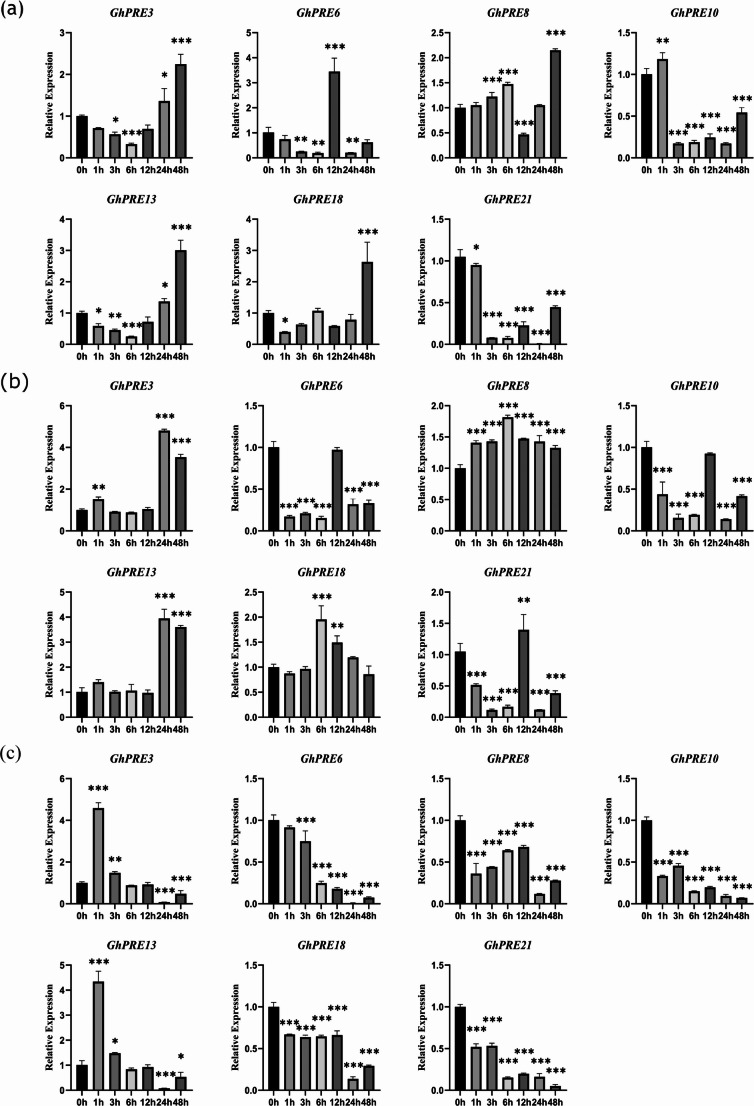
Expression levels of selected PRE key genes under abiotic stresses. (**a**) 350 mM NaCl solution treatment. (**b**) 20% PEG6000 solution is treated to simulate drought. (**c**) Low temperature incubator (4 ℃) simulates cold. Values are the means ± SD; *n* = 3. All statistical analyses were performed using Student’s t-test: *, *P* < 0.05; **, *P* < 0.01; ***, *P* < 0.001. All assays were repeated three times with similar results

To enhance our understanding of the PRE gene family’s role in tolerance to abiotic stress, we subjected TM-1 to three distinct abiotic stress treatments: drought, salt, and cold. We selected seven PRE genes from the TM-1 PRE gene family for real-time fluorescence quantitative expression analysis. Under salt stress conditions, the levels of expression for *GhPRE3*, *GhPRE13*, and *GhPRE18* initially showed a decrease, followed by an increase, with the expression levels at 48 h being significantly elevated compared to those at 0 h. In contrast, *GhPRE6* displayed a general downward trend in expression; nevertheless, it saw a significant rise at the 12-hour mark, exceeding the levels recorded at 0 h. On the other hand, while *GhPRE8* had an overall increasing trend, its expression level at 12 h was notably lower than at 0 h. The levels of expression for *GhPRE10* and *GhPRE21* displayed a declining trend, with the 48-hour levels significantly reduced compared to the 0-hour measurements (Fig. 7a). Under drought simulation conditions with 20% PEG6000, the expression levels for *GhPRE3* and *GhPRE13* initially remained stable but increased sharply after 24 h, reaching their maximum. The overall expression trends for *GhPRE6*, *GhPRE10*, and *GhPRE21* indicated considerable decreases; however, no significant differences were observed in the expression of *GhPRE6* and *GhPRE10* at 12 h relative to 0 h, while at 12 h, *GhPRE21*’s expression was greater than at other time points. *GhPRE8* followed an upward trajectory, with its highest expression observed at 6 h. During the initial phase, *GhPRE18*’s expression was stable, followed by a sharp rise to its peak at 6 h, and then a decrease, showing no significant change between 48 h and 0 h (Fig. 7b). In the context of cold treatment, the expression levels of *GhPRE3* and *GhPRE13* reached their peak at 1 h before starting to decline, whereas the expression levels of *GhPRE6*, *GhPRE10*, *GhPRE18*, and *GhPRE21* exhibited an overall decline. *GhPRE8* showed an initial decrease, a subsequent increase, and then a final decrease (Fig. 7c). It was also noted that the expression trends of *GhPRE3* and *GhPRE13* were almost identical, indicating a potential functional correlation between these two genes.

### Analysis of the subcellular localization of GhPRE3

**Fig. 8:**
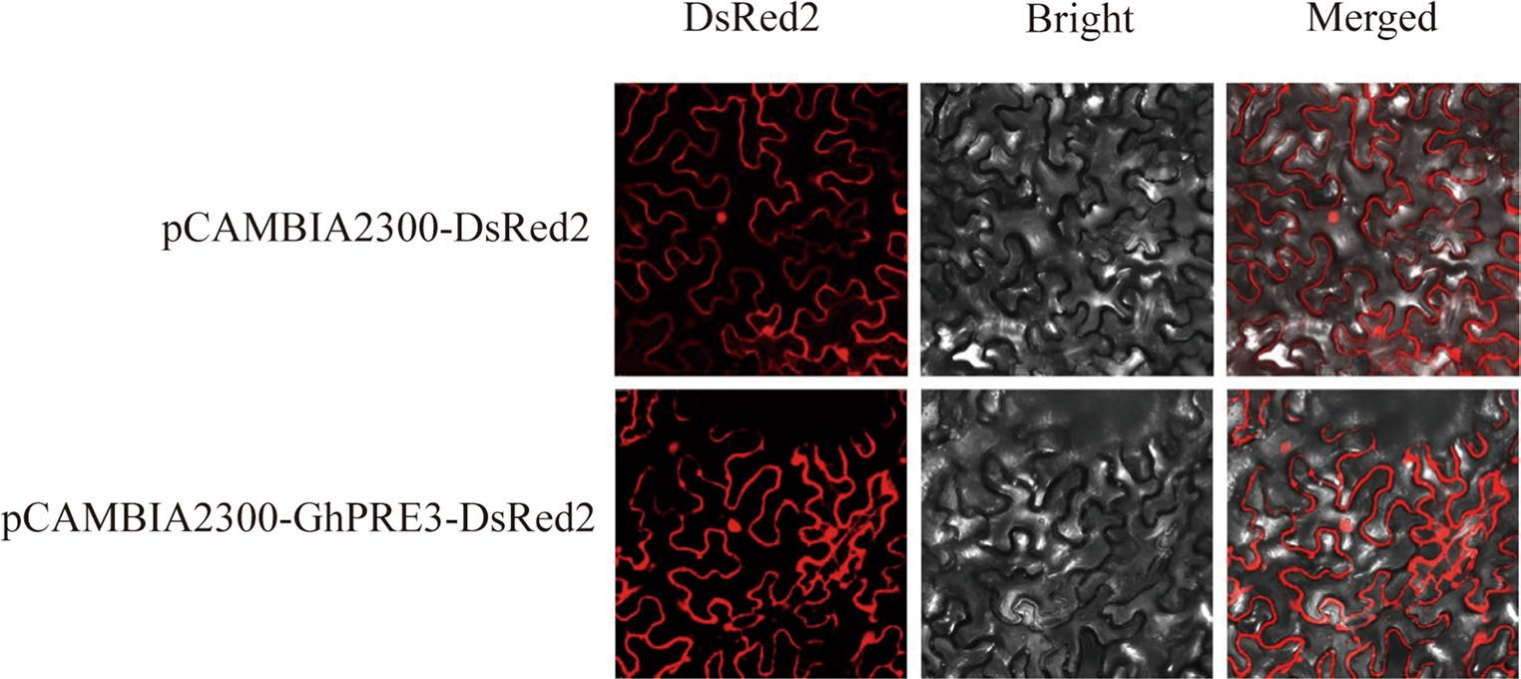
Subcellular localization of pCAMBIA2300-*GhPRE3*-DsRed2 fusion protein

To determine the subcellular localization of the *GhPRE3* gene, we constructed a fusion expression vector, pCAMBIA2300-GhPRE3-DsRed2, and introduced it into *Nicotiana benthamiana* plants infected with GV3101. As shown in Fig. 8, the empty vector pCAMBIA2300-DsRed2 exhibited strong fluorescence signals in the nucleus and cell membrane of *Nicotiana benthamiana* epidermal cells. Similarly, the pCAMBIA2300-GhPRE3-DsRed2 construct also displayed robust fluorescence in both the cell membrane and nucleus. This finding confirms the expression of the *GhPRE3* gene in cell membranes.

### Silencing of GhPRE3 in TM-1 reduced resistance to salinity stress and drought stress

**Fig. 9:**
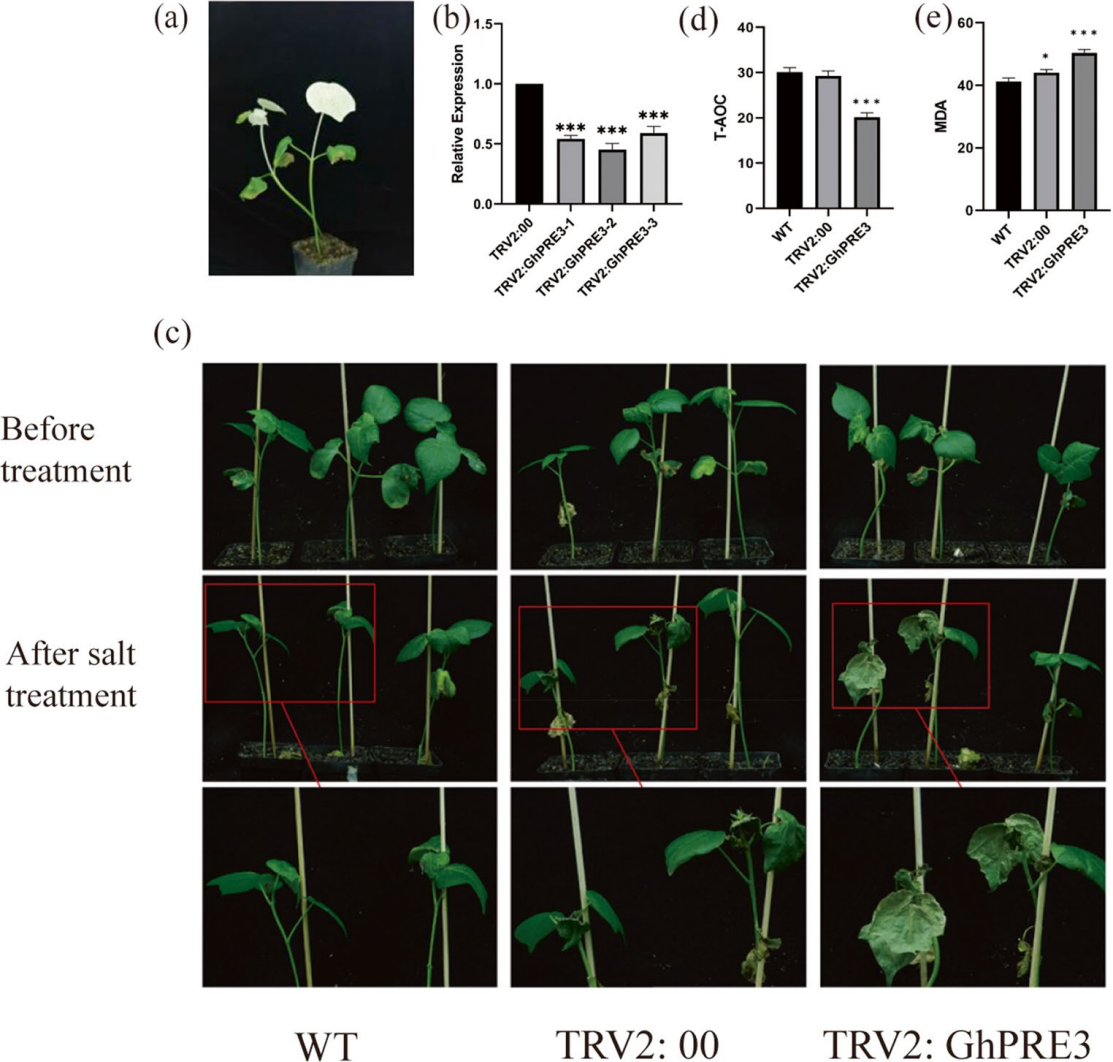
Silencing of *GhPRE3* gene by VIGS and analysis of T-AOC and MDA contents under salt stress. (**a**) PDS control plant. (**b**) *GhPRE3* silencing efficiency. (**c**) Phenotypes of WT, TRV2:00 and TRV2:*GhPRE3* under salt stress. (**d**, **e**) MDA and T-AOC determination of WT, TRV2:00 and TRV2:*GhPRE3*. Statistical significance with respect to the reference sample was determined by the student’s t-test: **, *p* < 0.01; ***, *p* < 0.001

Initially, the VIGS system demonstrated accuracy in observing leaf bleaching in cotton lines subjected to TRV2: PDS injections (Fig. 9a). To assess the expression levels of *GhPRE3* in the leaves of strains treated with TRV2: 00 and TRV2: *GhPRE3*, qRT-PCR was performed. This analysis indicated that the expression level of the *GhPRE3* gene in the silenced strains was significantly lower compared to the control plants, confirming successful gene silencing (Fig. 9b). When exposed to salt stress using a 350 mM NaCl solution, the severity of leaf wilting in the gene-silenced plants was markedly greater than thay in the wild type (WT) and TRV2:00 plants (Fig. 9c). Additional evaluations through total antioxidant capacity (T-AOC) and malondialdehyde (MDA) analysis revealed that the T-AOC in the TRV2:*GhPRE3* strain was significantly lower than in the WT and TRV2:00 strains, while the MDA content in the TRV2:*GhPRE3* strain was considerably higher than that in the control groups (Fig. 9d, e). These findings suggest that the silenced expression of *GhPRE3* reduced the antioxidant capacity in cotton and diminished its tolerance to salt stress.

**Fig. 10:**
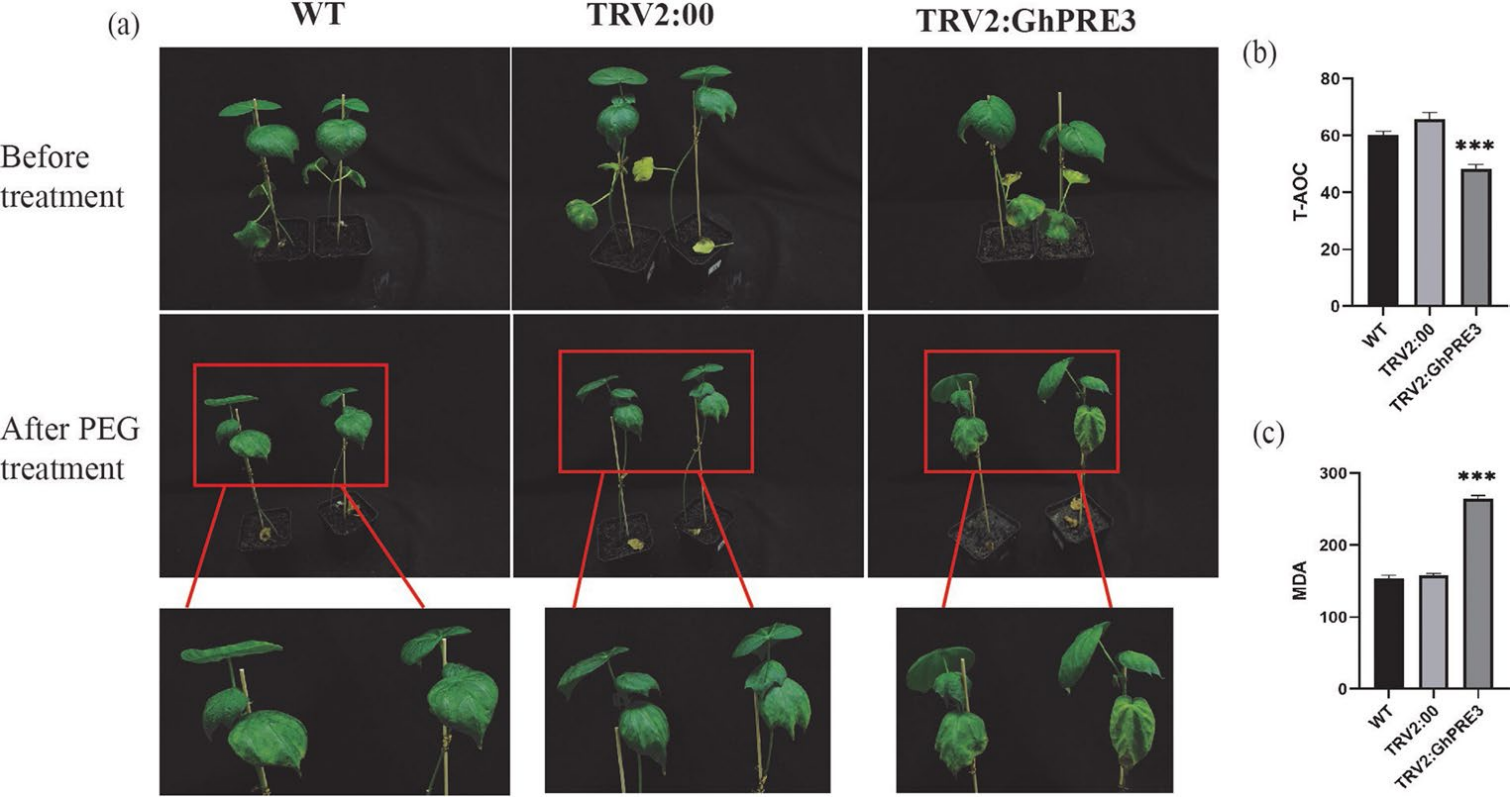
Silencing of *GhPRE3* gene by VIGS and analysis of T-AOC and MDA contents under drought stress. (**a**) Phenotypes of WT, TRV2:00 and TRV2:*GhPRE3* under drought stress. (**b**, **c**) MDA and T-AOC determination of WT, TRV2:00 and TRV2:*GhPRE3*. Statistical significance with respect to the reference sample was determined by the student’s t-test: **, *p* < 0.01; ***, *p* < 0.001

To verify the effect of gene *GhPRE3* on drought stress in cotton, another VIGS experiment was conducted. Cotton leaves injected with TRV2: PDS showed chlorosis, and the expression level of *GhPRE3* in cotton injected with TRV2:*GhPRE3* was significantly reduced (Fig. S1 a, b). After treating with 20% PEG6000 to simulate drought conditions, it was found that cotton with down-regulated *GhPRE3* showed faster leaf wilting (Fig. 10a), weaker total antioxidant capacity (T-AOC) (Fig. 10b), and higher content of oxidative product malondialdehyde (MDA) (Fig. 10c) compared to the WT cotton. These results indicate that down-regulation of the cotton gene *GhPRE3* reduced salt and drought tolerance, and *GhPRE3* positively participates in cotton salt and drought resistance pathways in cotton.

### Overexpression of GhPRE3 enhanced seed germination and root growth of Arabidopsis thaliana under abiotic stress

**Fig. 11:**
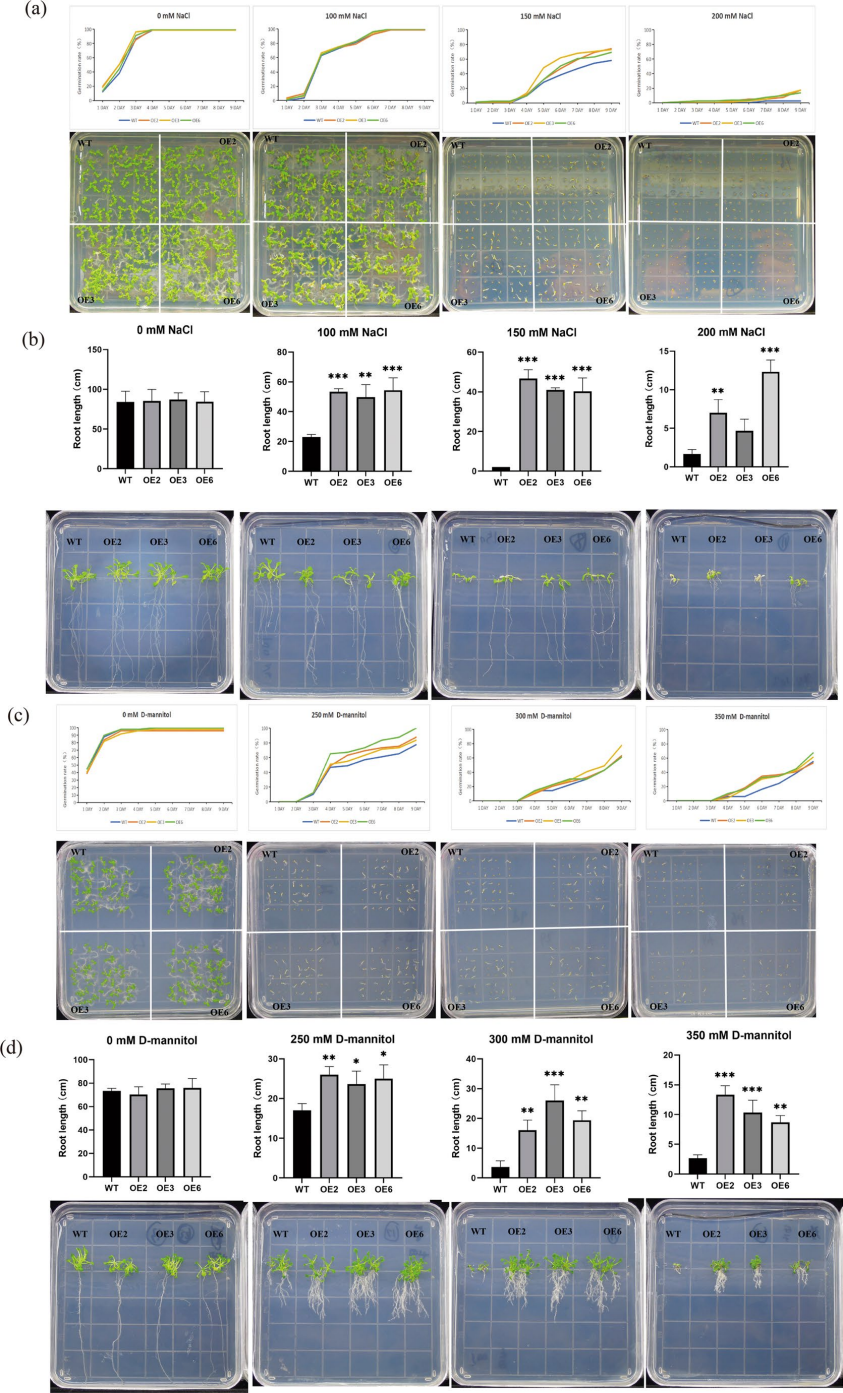
Overexpression of *GhPRE3* enhanced seed germination and root length of *Arabidopsis thaliana* under salt stress and drought stress during germination period. (**a**, **b**) Germination rate and root length of WT *Arabidopsis thaliana* and *GhPRE3* overexpressing Arabidopsis under different salt concentrations (0 mM, 100 mM, 150 mM, 200 mM). (**c**, **d**) Germination rate and root length of WT Arabidopsis thaliana and *GhPRE3* overexpressing Arabidopsis under different D-mannitol concentrations (0 mM, 250 mM, 300 mM, 350 mM). OE2, OE3, and OE6 represent three biological replicates, with each concentration test having three technical replicates. Statistical significance with respect to the reference sample was determined by the student’s t-test: **, *p* < 0.01; ***, *p* < 0.001

We confirmed through PCR tests that the transgenic *Arabidopsis* plants were positive (Fig. S2 a). Three *Arabidopsis thaliana* transgenic lines (OE2, OE3, and OE6) with relatively high expression levels of *GhPRE3* were sown on media containing different concentrations of salt (100 mM, 150 mM, 200 mM) and mannitol (250 mM, 300 mM, 350 mM) (Fig. S2 b). Under salt treatment, there was no significant difference in germination rate between the overexpression lines (OE2, OE3, OE6) and the WT line in 0 mM and 100 mM media. At 150 mM, the germination rates of all three overexpression lines were significantly higher than that of the WT, with the most obvious difference on the 7th day. Under 200 mM salt treatment, the germination rates of all *Arabidopsis* lines were inhibited, but on the 8th and 9th days, the germination rates of the overexpression lines were significantly higher than that of the WT. We also observed root length under salt treatment. In 0 mM medium, there was no difference in root length between the WT and overexpression lines. In 100 mM, 150 mM, and 200 mM media, the root lengths of the three overexpression lines were significantly longer than that of the WT, although the overexpression lines were also significantly inhibited at 200 mM (Fig. 11a, b). Under drought treatment simulated by mannitol, we found that the *GhPRE3* overexpression lines also showed stress resistance. In 0 mM mannitol, there was no difference in germination rate and root length between the overexpression lines and WT lines. At 250 mM, the germination rate and root length of the overexpression lines were significantly higher than those of the WT. At 300 mM, the germination rate of the overexpression lines was slightly higher than that of the WT, while the root length of the WT line was severely inhibited; the root length of the overexpression lines was about four times that of the WT. At 350 mM, the difference in germination rate was most obvious, with the largest difference between the overexpression lines and WT on the 6th day. Root length observations showed that the roots of the overexpression lines were longer than those of the WT, and although root length decreased with increasing concentration, lateral root hairs increased (Fig. 11c, d). In summary, overexpression of *GhPRE3* improved *Arabidopsis* resistance to salt and drought stress during seed germination.

## Discussion

Cotton has emerged as a vital cash crop in agriculture, serving as a significant raw material for the textile sector. Enhancing both the yield and quality of cotton is essential for fostering economic growth and improving human well-being. Throughout the lengthy course of biological evolution, plants have developed various stress-resistance mechanisms, including osmotic regulation, scavenging of reactive oxygen species, and metabolic adjustments. Signaling processes are significantly influenced by transcription factors, with the basic helix-loop-helix (bHLH) family being one of the largest gene families identified in *Arabidopsis thaliana*, characterized by a highly conserved bHLH motif across eukaryotes. This gene family encompasses two well-conserved elements: a fundamental DNA binding domain and a helix-loop-helix (HLH) segment (Hao et al. 2021). In tomato (*Solanum lycopersicum* L.), the transcription factor *SlbHLH92* regulates the expression of the L-Cysteine DESULFHYDRASE 1 (*SlLCD1*) gene, which is involved in the production of hydrogen sulfide (H2S), a gaseous signaling molecule that enhances the plant resistance to salt stress (Lu et al. 2024). Furthermore, the overexpression of the *CabHLH035* protein in pepper (*Capsicum annuum* L.) improved salt tolerance in both pepper and *Arabidopsis*, whereas the knockout of *CabHLH035* results in reduced salt resistance in these two plant species (Zhang et al. 2022).

As a subgroup of bHLH transcription factors, PRE transcription factors play a significant role in the processes related to plant growth, development, and stress resilience. The literature review indicates a scarcity of studies focusing on PRE genes. In our research, we identified a total of 68 PRE genes across four cotton species, which were categorized into three subgroups (Fig. 1). Through motif analysis, structural analysis, collinearity analysis, and other methodologies, we observed that PRE genes exhibit high conservation throughout the polyploidy copying and evolutionary processes in cotton (Figs. 2 and 4). This conservation may explain the limited size of the PRE gene family in cotton (Fig. 3).

In the functional analysis study of the PRE subfamily, it was discovered that PRE proteins are involved in the signal transduction pathways related to hormones, temperature, and light responses, thereby regulating plant growth and development through various mechanisms. Specifically, they play a role in cell elongation in response to gibberellin, brassinolide, auxin, and light signals in plants (Chapman et al. 2012; Hyun and Lee 2006 ; Lee et al. 2006; Li et al. 2006; Wang et al. 2009; Zhu et al. 2017). PRE1 was localized in the nucleus of *Arabidopsis thaliana*, where it was found to inhibit gibberellin biosynthesis, contributing to the dwarf phenotype observed in mutants. This suggests that PRE1 is involved in gibberellin-regulated cell elongation (Zhang et al. 2009). In tomato (*Lycopersicon esculentum*), SlPRE2 has been identified as a regulator of style length and has been shown to influence plant morphological changes by modulating the activity of bHLH proteins involved in light signaling (Chen et al. 2007). Furthermore, it acts as a negative regulator of chlorophyll and carotenoid accumulation in fruit. Cis-regulatory element analysis of cotton PRE genes revealed the presence of various regulatory elements associated with different response pathways, including light response, gibberellin response, and abscisic acid response (Fig. 5). This finding supports previous reports that PRE genes are involved in plant growth and gibberellin regulation pathways.

PRE gene family plant an important role in plant responses to abiotic stresses such as low temperature, drought, and emergency conditions. Certain SbPRE genes in Sorghum (*Sorghum bicolor*) were activated by abiotic stress and aphid infection, with *SbPRE4* enhancing Sorghum’s resistance to aphids through the accumulation of jasmonic acids (JAs) (Guo et al. 2024). Additionally, a study involving apple (*Malus domestica*) revealed that *MdPRE4.3* reduces the apple’s tolerance to NaCl, abscisic acid (ABA), and indoleacetic acid (IAA) (Li et al. 2022). Populus aspens overexpressing *PsPRE1* exhibited a reduction in oxidative processes under salt stress by enhancing catalase (CAT) activity, thereby improving salt tolerance (Du et al. 2023). Within the cotton genome, PRE1 has been identified as a positive enhancer of fiber cell elongation; overexpression of *GhPRE1* leads to longer cotton fibers and improved fiber quality (Zhao et al. 2018). By downloading cotton PRE gene expression data from the cotton database and generating heat maps, we found that GhPRE genes showed significant differential expression under abiotic stress, especially *GhPRE3* (Fig. 6).

In this study, we studied PRE gene expression in TM-1 under salt, drought, and cold stress conditions. We found that the expression levels of PRE genes showed significant changes with the increase of abiotic stress treatment time, indicating that PRE genes may be involved in cotton’s response pathways to abiotic stress (Fig. 7). To further confirm the response of PRE genes to abiotic stress, we selected *GhPRE3* as our target gene. Cotton VIGS experiments confirmed that the expression of *GhPRE3* was down-regulated in the experimental lines compared to wild-type cotton. Through stress treatments of both experimental and wild-type cotton, we observed that the experimental lines showed more severe leaf wilting under both 350 mM salt solution treatment and 20% PEG6000 solution treatment. Meanwhile, we corroborated this result by measuring the contents of T-AOC and MDA. T-AOC serves as an indicator of plant antioxidant capacity. After the downregulation of *GhPRE3* gene, the antioxidant capacity of cotton leaves decreased, resulting in a significant increase in the content of oxidative product MDA compared to the untreated wild type (Fig. 9, Fig, 10). As a model plant, *Arabidopsis thaliana* is commonly used in various validation experiments. By constructing Arabidopsis lines overexpressing *GhPRE3*, we conducted simulated experiments of salt and drought treatments on Arabidopsis during the germination period. We found that overexpression of *GhPRE3* enhanced the germination rate and root length of Arabidopsis, indicating that *GhPRE3* plays a positive role in salt and drought resistance (Fig. 11). We also confirmed through subcellular localization experiments that *GhPRE3* is a membrane-bound protein (Fig. 8). Cell membranes play an indispensable role in plant signal transduction. Regarding the abiotic stress resistance mechanism of *GhPRE3*, we believe it is related to membrane signal transduction, which will be the focus of our future research.

Through cotton VIGS experiments and Arabidopsis overexpression experiments, it has been proven that the GhPRE3 gene plays a positive role in cotton’s salt and drought resistance. With the quick development of genetic technologies and tools, specific CRISPR/Cas genome editing and transgenic overexpression (Chen et al. 2017; Li et al. 2024, 2025; Ribeiro et al. 2021), the functions of these genes will be finally elucidated and will be used to enhance plants’ specific traits, such as yield, quality and resistance to a variety of environmental stresses. The screening and analysis of cotton PRE genes have filled the gap in relevant research, especially the discovery of GhPRE3, which provides a reference gene for cotton genetic improvement.

## Conclusions

In this research, a total of 68 PRE genes were identified from four different cotton species. These genes were categorized into three subgroups according to their evolutionary relationships. By conducting physicochemical analysis, MOTIF analysis, and examining gene structures, we demonstrated that PRE genes exhibit a high level of conservation throughout cotton evolution. Collinear analysis revealed that the mechanisms driving the evolution of PRE genes primarily involve gene duplication and expansion. Additionally, an analysis of promoter cis-acting elements and heat map evaluations led to the identification of seven representative GhPRE genes for qRT-PCR analysis, showing notable variations in gene expression in response to abiotic stresses such as salinity, drought, and cold temperatures. Studies on the subcellular localization of the *GhPRE3* gene confirmed its presence in membrane structures. Furthermore, VIGS experiments illustrated that *GhPRE3* plays a positive role in enhancing salt and drought tolerance in cotton. Overexpression of *GhPRE3* also enhances the germination rate and root length of *Arabidopsis thaliana* under salt and drought stress during germination. These findings offer significant insights for the genetic enhancement and breeding efforts in cotton.

## Supplementary Information

Below is the link to the electronic supplementary material.

Supplementary Material 1

## Data Availability

Data is provided within the manuscript or supplementary information files.
