# Supplementary material for: Genome-wide identification and analysis of paclobutrazol-resistance gene family in cotton and the positive role of GhPRE3 in salt stress and drought stress resistance
Source: Funct Integr Genomics. 2025 Sep 29;25(1):201. doi: 10.1007/s10142-025-01701-2 (PMC12479684; doi:10.1007/s10142-025-01701-2)
Supplement: Supplementary file 1 — Supplementary Material 1 [file 10142_2025_1701_MOESM1_ESM.doc]

Supplementary to

Genome-wide identification and analysis of paclobutrazol-resistance gene family in cotton and the positive role of *GhPRE3* in salt stress and drought stress resistance

**Fig.**
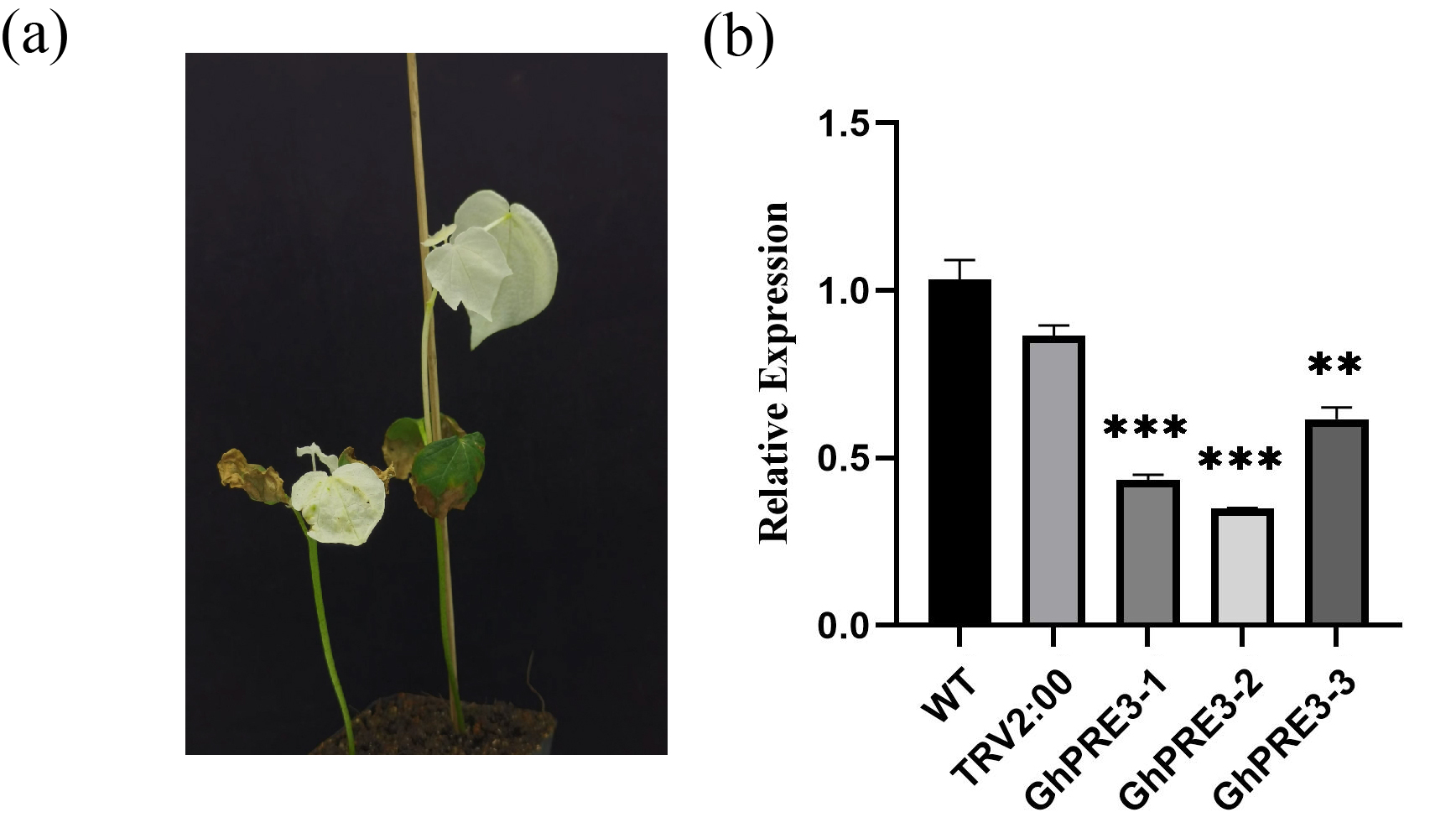
**S1.** Verification of the downregulation of the *GhPRE3* gene in the VIGS experiment. (**a**) PDS control plant. (**b**) *GhPRE3* silencing efficiency. Statistical significance with respect to the reference sample was determined by the student’s t-test: **, p < 0.01; ***, p < 0.001.

**Fig. S2**. Positive validation of overexpression of *GhPRE3* in Arabidopsis thaliana
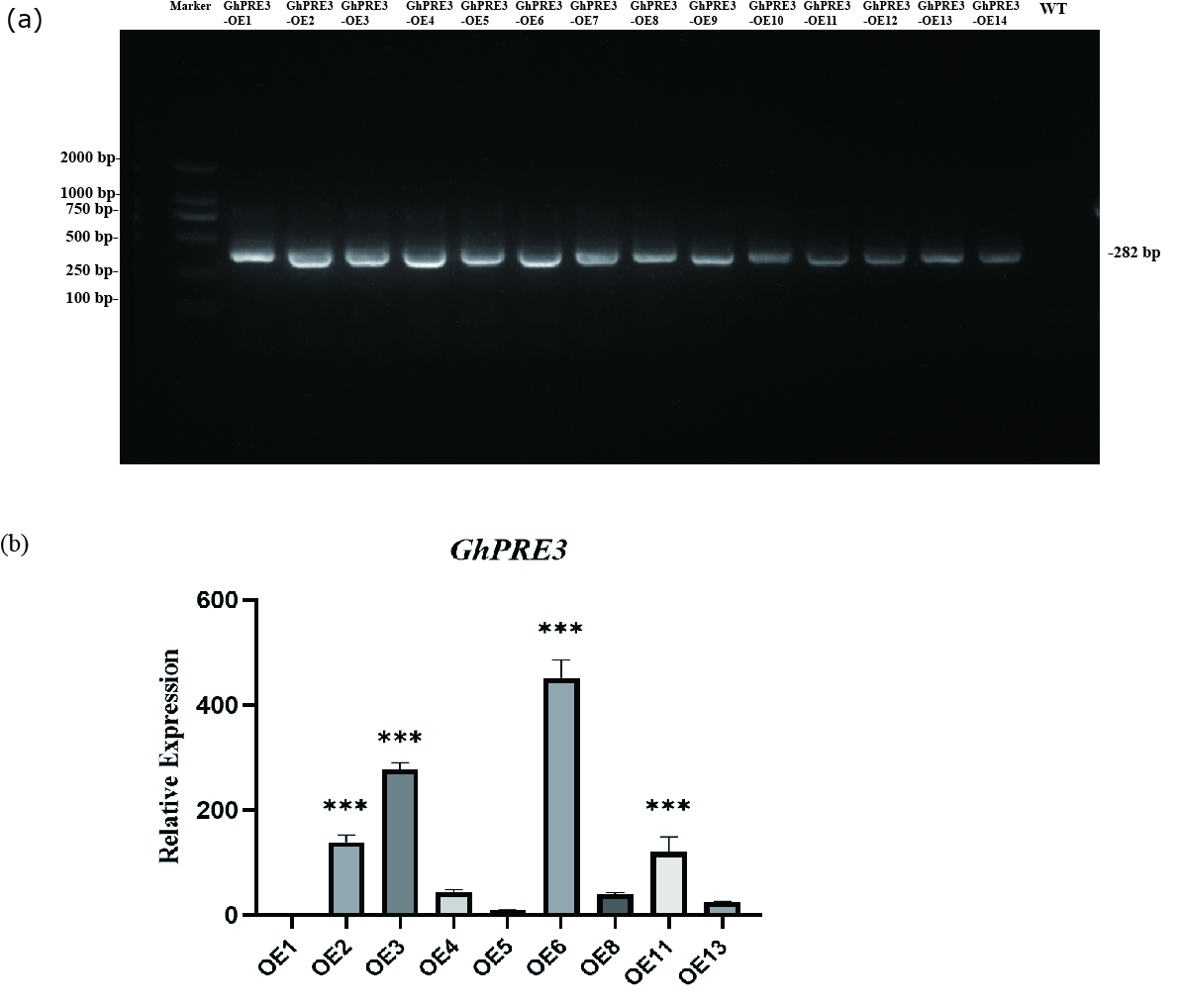
. (a) Electrophoresis results of PCR amplification of overexpressed Arabidopsis thaliana lines' DNA. (b) Relative expression levels of *GhPRE3* gene among overexpressed Arabidopsis lines. Statistical significance with respect to the reference sample was determined by the student’s t-test: **, p < 0.01; ***, p < 0.001.

Table S1. The expression profiles of PRE genes in cotton under different treatment conditions

| gene ID | gene | TM1_0h | 4℃_1h | 4℃_3h | 4℃_6h | 4℃_12h | 4℃_24h | 37℃_1h | 37℃_3h | 37℃_6h | 37℃_12h |
| --- | --- | --- | --- | --- | --- | --- | --- | --- | --- | --- | --- |
| Ghi_A03G04046 | GhPRE1 | 0 | 0 | 0 | 0 | 0 | 0 | 0 | 0 | 0 | 0 |
| Ghi_A03G06271 | GhPRE2 | 0.28 | 0 | 0.04 | 0 | 0 | 0 | 0.2 | 0 | 0 | 0 |
| Ghi_A03G06956 | GhPRE3 | 5.61 | 5.29 | 4.9 | 5.54 | 4.47 | 1.2 | 0.08 | 4.85 | 2.28 | 1.85 |
| Ghi_A05G17911 | GhPRE4 | 0.65 | 0 | 0.35 | 0.16 | 0 | 0 | 0 | 0.47 | 0 | 0 |
| Ghi_A05G21651 | GhPRE5 | 0 | 0.09 | 0 | 0 | 0 | 0 | 0 | 0 | 0 | 0 |
| Ghi_A07G13076 | GhPRE6 | 0.21 | 0.19 | 0.26 | 0.78 | 0 | 0 | 0.25 | 0 | 0.32 | 0 |
| Ghi_A09G01046 | GhPRE7 | 0 | 0 | 0.15 | 0 | 0 | 0 | 0.06 | 0 | 0 | 0 |
| Ghi_A09G01061 | GhPRE8 | 2.6 | 2.68 | 2.37 | 2.45 | 1.87 | 1.61 | 2.39 | 3.13 | 2.83 | 2.1 |
| Ghi_A11G01796 | GhPRE9 | 0 | 0 | 0 | 0 | 0 | 0 | 0 | 0 | 0 | 0 |
| Ghi_A12G10041 | GhPRE10 | 0.27 | 0.1 | 0.08 | 0 | 0.53 | 0 | 0 | 1.27 | 0.39 | 0.51 |
| Ghi_A12G10521 | GhPRE11 | 0 | 0 | 0 | 0 | 0 | 0 | 0 | 0 | 0 | 0 |
| Ghi_A13G03981 | GhPRE12 | 0.05 | 0.06 | 0 | 0 | 0 | 0 | 0 | 0 | 0 | 0 |
| Ghi_D02G06716 | GhPRE13 | 4.39 | 2.54 | 2.74 | 3.36 | 1 | 1.63 | 0.18 | 3.75 | 0.76 | 1.07 |
| Ghi_D03G05676 | GhPRE14 | 0 | 0 | 0 | 0 | 0 | 0 | 0 | 0 | 0 | 0 |
| Ghi_D04G02451 | GhPRE15 | 0 | 0 | 0 | 0 | 0 | 0 | 0 | 0 | 0 | 0 |
| Ghi_D07G12231 | GhPRE16 | 0 | 0 | 0 | 0 | 0 | 0 | 0.09 | 0.04 | 0 | 0.08 |
| Ghi_D09G01021 | GhPRE17 | 0 | 0 | 0 | 0 | 0 | 0.07 | 0 | 0 | 0 | 0 |
| Ghi_D09G01031 | GhPRE18 | 1.91 | 2.49 | 2.08 | 1.17 | 2.37 | 1.51 | 1.32 | 2.85 | 2.36 | 2.36 |
| Ghi_D11G01701 | GhPRE19 | 0 | 0 | 0 | 0 | 0 | 0 | 0 | 0 | 0 | 0 |
| Ghi_D11G01706 | GhPRE20 | 0 | 0 | 0 | 0 | 0 | 0 | 0 | 0 | 0 | 0 |
| Ghi_D12G10716 | GhPRE21 | 0 | 0.22 | 0.32 | 0.11 | 0 | 0 | 0 | 1.56 | 0.06 | 0.23 |
| Ghi_D12G11236 | GhPRE22 | 0 | 0 | 0 | 0 | 0 | 0 | 0.1 | 0 | 0 | 0 |
| Ghi_D13G03911 | GhPRE23 | 0 | 0 | 0.11 | 0 | 0 | 0 | 0 | 0.09 | 0 | 0 |
|  |  |  |  |  |  |  |  |  |  |  |  |
| gene | 37℃_24h | NaCl_1h | NaCl_3h | NaCl_6h | NaCl_12h | NaCl_24h | PEG_1h | PEG_3h | PEG_6h | PEG_12h | PEG_24h |
| GhPRE1 | 0 | 0 | 0 | 0 | 0 | 0 | 0 | 0 | 0 | 0 | 0 |
| GhPRE2 | 0 | 0 | 0 | 0.23 | 0 | 0 | 0.14 | 0 | 0.36 | 0.89 | 0.04 |
| GhPRE3 | 3.74 | 1.91 | 4.33 | 5.51 | 4.11 | 5.06 | 4.14 | 3.68 | 3.45 | 5.54 | 6.13 |
| GhPRE4 | 0 | 0 | 0 | 0.65 | 0.8 | 0 | 0.64 | 0 | 0.9 | 1.36 | 0.08 |
| GhPRE5 | 0 | 0 | 0 | 0 | 0 | 0 | 0 | 0 | 0 | 0 | 0.04 |
| GhPRE6 | 0.23 | 0.3 | 0 | 0.34 | 0.57 | 0.15 | 0 | 0.16 | 0 | 1.28 | 0 |
| GhPRE7 | 0 | 0 | 0 | 0.55 | 0.17 | 0 | 0 | 0 | 0 | 0 | 0.08 |
| GhPRE8 | 3.02 | 2.69 | 1.92 | 2.64 | 4.45 | 1.65 | 2.54 | 2.17 | 1.92 | 4.9 | 3.44 |
| GhPRE9 | 0 | 0 | 0 | 0 | 0 | 0 | 0 | 0 | 0 | 0 | 0 |
| GhPRE10 | 0 | 0.34 | 0.54 | 0.46 | 0.12 | 0.57 | 0.75 | 0.07 | 0 | 0.78 | 0.85 |
| GhPRE11 | 0 | 0 | 0 | 0 | 0 | 0 | 0 | 0 | 0 | 0 | 0 |
| GhPRE12 | 0 | 0.09 | 0 | 0 | 0 | 0.04 | 0 | 0 | 0 | 0 | 0.14 |
| GhPRE13 | 0.42 | 0.29 | 2.87 | 3.9 | 3.51 | 2.02 | 2.78 | 1.89 | 2.82 | 5.3 | 3.73 |
| GhPRE14 | 0 | 0 | 0 | 0 | 0 | 0 | 0 | 0 | 0 | 0 | 0 |
| GhPRE15 | 0 | 0 | 0 | 0 | 0 | 0 | 0 | 0 | 0 | 0 | 0 |
| GhPRE16 | 0 | 0 | 0 | 0.05 | 0.26 | 0 | 0 | 0 | 0 | 0.29 | 0 |
| GhPRE17 | 0 | 0 | 0 | 0 | 0 | 0 | 0 | 0 | 0 | 0 | 0 |
| GhPRE18 | 1.88 | 1.52 | 1.59 | 2.06 | 4.51 | 1.82 | 1.91 | 1.44 | 1.35 | 4.63 | 2.9 |
| GhPRE19 | 0 | 0 | 0 | 0 | 0 | 0 | 0 | 0 | 0 | 0 | 0 |
| GhPRE20 | 0 | 0 | 0 | 0 | 0 | 0 | 0 | 0 | 0 | 0 | 0 |
| GhPRE21 | 0 | 0 | 0.48 | 0.26 | 0.85 | 0.16 | 0.87 | 0 | 0 | 0.65 | 0.75 |
| GhPRE22 | 0 | 0 | 0 | 0 | 0 | 0 | 0 | 0 | 0 | 0 | 0 |
| GhPRE23 | 0 | 0 | 0 | 0 | 0 | 0 | 0.23 | 0 | 0 | 0.05 | 0 |

Table S2. Primers used in qRT-PCR analysis, Subcellular localization assay and VIGS assay

| Primer name |  | Primer (5'-3') | Usage |
| --- | --- | --- | --- |
| GhActin7 | Forward | ATCCTCCGTCTTGACCTTG | Reference gene of cotton qRT-PCR |
|  | Reverse | TGTCCGTCAGGCAACTCAT |  |
| GhPRE3 | Forward | GCTGCAACACCTTATCCCTG | qRT-PCR of *GhPRE3* transcripts |
|  | Reverse | CGCCGTCTGTGGAAGCTAA |  |
| GhPRE6 | Forward | TGTCTCCAAGTTACGCCACC | qRT-PCR of *GhPRE6* transcripts |
|  | Reverse | AAGCTGAGAGAGTCGGTCAC |  |
| GhPRE8 | Forward | ACAGTCAGCAGCAGGTGTTT | qRT-PCR of *GhPRE8* transcripts |
|  | Reverse | GCCTATCCCGAATCTCAGGC |  |
| GhPRE10 | Forward | CACGTTCCAGGCAATCAGGT | qRT-PCR of *GhPRE10* transcripts |
|  | Reverse | CTGATACCTTGTCGGAGCGT |  |
| GhPRE13 | Forward | AGCTGCAACACCTTATCCCT | qRT-PCR of *GhPRE13* transcripts |
|  | Reverse | CGCTATCGCTGTCTGTGGAA |  |
| GhPRE18 | Forward | TCGAGACAGTCAACAGCAGG | qRT-PCR of *GhPRE18* transcripts |
|  | Reverse | GAGCCGTTCACTTAGGTCGT |  |
| GhPRE21 | Forward | CGTTCTAGGCAATCAGGTGC | qRT-PCR of *GhPRE21* transcripts |
|  | Reverse | CGATCGCTTAAGCCGTCAAC |  |
| GhPRE3-RFP | Forward | cctcgagcgggggactctagaATGTCAGG  CAGAAGATCACGTTCC | Subcellular localization of *GhPRE3* |
|  | Reverse | catggtaccccccggggatccTTGCATAAGTA  AACTCCTTATAATGGC |  |
| GhPRE3-2300 | Forward | gcgggggactctagaggatccATGTCAGGCAGAAGATCACG | Primers for Arabidopsis thaliana Overexpression Vector Construction |
|  | Reverse | catggtaccccccggggatccTTGCATAAGTAAACTCCTTA |  |
| GhPRE3-VIGS | Forward | aggttaccgaattctctagaATGTCAGGCA  GAAGATCACG | virus induced gene silencing of *GhPRE3* |
|  | Reverse | agacgcgtgagctcggtaccTTGGTCGCT  ACCGCCGTCTG |  |
| AtActin | Forward | TTGTGCTGGATTCTGGTGATGG | Arabidopsis thaliana qRT-PCR reference primers |
|  | Reverse | CCGCTCTGCTGTTGTGGTG |  |

Table S3. Physicochemical properties of PRE subfamily genes in cotton

| Gene name | Gene ID | Number of amino acids | Molecular weight | Theoretical pI | Instability index | Aliphatic index | Grand average of hydropathicity (GRAVY) | subcellular localization |
| --- | --- | --- | --- | --- | --- | --- | --- | --- |
| GaPRE1 | Gar01G22470 | 91 | 10359.66 | 9.03 | 81.61 | 94.29 | -0.592 | mito: |
| GaPRE2 | Gar03G14630 | 93 | 10616.99 | 9.50 | 85.80 | 94.30 | -0.612 | nucl: |
| GaPRE3 | Gar04G05860 | 91 | 10330.60 | 6.40 | 68.73 | 96.59 | -0.486 | mito: |
| GaPRE4 | Gar05G34470 | 93 | 10570.07 | 9.99 | 73.93 | 107.96 | -0.439 | nucl: |
| GaPRE5 | Gar07G28400 | 94 | 10453.85 | 7.95 | 81.55 | 105.85 | -0.335 | mito: |
| GaPRE6 | Gar09G02640 | 92 | 10412.85 | 7.94 | 81.36 | 108.15 | -0.371 | mito: |
| GaPRE7 | Gar11G03510 | 92 | 10419.67 | 7.98 | 68.43 | 95.43 | -0.614 | mito: |
| GaPRE8 | Gar11G03530 | 89 | 10085.38 | 5.25 | 62.70 | 105.17 | -0.469 | cyto_nucl: |
| GaPRE9 | Gar12G21840 | 92 | 10344.69 | 9.61 | 77.84 | 98.59 | -0.537 | nucl: |
| GaPRE10 | Gar12G22940 | 87 | 9743.08 | 5.56 | 55.51 | 102.07 | -0.395 | nucl: |
| GaPRE11 | Gar13G08900 | 89 | 9865.21 | 6.82 | 64.80 | 101.91 | -0.352 | nucl: |
| GbPRE1 | GB_A03G0781 | 91 | 10359.66 | 9.03 | 81.61 | 94.29 | -0.592 | mito: |
| GbPRE2 | GB_A03G1213 | 93 | 10558.96 | 9.84 | 85.06 | 94.30 | -0.578 | nucl: |
| GbPRE3 | GB_A05G3156 | 93 | 10629.14 | 10.35 | 74.24 | 107.96 | -0.470 | mito: |
| GbPRE4 | GB_A05G3944 | 91 | 10330.60 | 6.40 | 68.73 | 96.59 | -0.486 | mito: |
| GbPRE5 | GB_A07G2518 | 94 | 10463.89 | 7.95 | 78.93 | 105.85 | -0.344 | mito: |
| GbPRE6 | GB_A09G0251 | 93 | 10540.62 | 9.57 | 89.34 | 73.44 | -1.147 | nucl: |
| GbPRE7 | GB_A09G0253 | 92 | 10412.85 | 7.94 | 81.36 | 108.15 | -0.371 | mito: |
| GbPRE8 | GB_A11G0334 | 92 | 10419.67 | 7.98 | 68.43 | 95.43 | -0.614 | mito: |
| GbPRE9 | GB_A12G1933 | 92 | 10344.69 | 9.61 | 77.84 | 98.59 | -0.537 | nucl: |
| GbPRE10 | GB_A12G2038 | 87 | 9729.01 | 5.18 | 58.82 | 102.07 | -0.391 | nucl: |
| GbPRE11 | GB_D02G1393 | 93 | 10648.00 | 9.03 | 88.69 | 94.30 | -0.616 | nucl: |
| GbPRE12 | GB_D03G1072 | 91 | 10359.66 | 9.03 | 81.61 | 94.29 | -0.600 | nucl: |
| GbPRE13 | GB_D04G0515 | 91 | 10294.70 | 7.98 | 68.28 | 105.16 | -0.391 | mito: |
| GbPRE14 | GB_D05G3131 | 93 | 10589.12 | 10.35 | 71.39 | 107.96 | -0.445 | nucl: |
| GbPRE15 | GB_D07G2497 | 94 | 10483.93 | 7.95 | 72.69 | 105.85 | -0.291 | mito: |
| GbPRE16 | GB_D09G0218 | 101 | 11442.55 | 9.79 | 91.75 | 67.62 | -1.242 | nucl: |
| GbPRE17 | GB_D09G0221 | 92 | 10384.79 | 6.57 | 82.81 | 108.15 | -0.360 | mito: |
| GbPRE18 | GB_D11G0338 | 92 | 10419.67 | 7.98 | 68.43 | 95.43 | -0.614 | mito: |
| GbPRE19 | GB_D11G0339 | 89 | 9977.29 | 5.20 | 71.11 | 105.17 | -0.328 | nucl: |
| GbPRE20 | GB_D12G1921 | 92 | 10354.73 | 9.61 | 74.34 | 98.59 | -0.546 | nucl: |
| GbPRE21 | GB_D12G2029 | 87 | 9678.90 | 5.56 | 53.36 | 96.44 | -0.444 | nucl: |
| GbPRE22 | GB_D13G0771 | 89 | 9895.17 | 6.25 | 61.90 | 101.91 | -0.344 | nucl: |
| GhPRE1 | Ghi_A03G04046 | 91 | 10359.66 | 9.03 | 81.61 | 94.29 | -0.592 | mito: |
| GhPRE2 | Ghi_A03G06271 | 93 | 10570.07 | 9.99 | 71.86 | 107.96 | -0.431 | nucl: |
| GhPRE3 | Ghi_A03G06956 | 93 | 10558.96 | 9.84 | 85.06 | 94.30 | -0.578 | nucl: |
| GhPRE4 | Ghi_A05G17911 | 93 | 10570.07 | 9.99 | 73.93 | 107.96 | -0.439 | nucl: |
| GhPRE5 | Ghi_A05G21651 | 91 | 10330.60 | 6.40 | 68.73 | 96.59 | -0.486 | mito: |
| GhPRE6 | Ghi_A07G13076 | 94 | 10463.89 | 7.95 | 78.93 | 105.85 | -0.344 | mito: |
| GhPRE7 | Ghi_A09G01046 | 93 | 10540.62 | 9.57 | 89.34 | 73.44 | -1.147 | nucl: |
| GhPRE8 | Ghi_A09G01061 | 92 | 10382.82 | 7.94 | 81.36 | 109.24 | -0.343 | mito: |
| GhPRE9 | Ghi_A11G01796 | 92 | 10419.67 | 7.98 | 68.43 | 95.43 | -0.614 | mito: |
| GhPRE10 | Ghi_A12G10041 | 92 | 10344.69 | 9.61 | 77.84 | 98.59 | -0.537 | nucl: |
| GhPRE11 | Ghi_A12G10521 | 87 | 9729.01 | 5.18 | 58.82 | 102.07 | -0.391 | nucl: |
| GhPRE12 | Ghi_A13G03981 | 89 | 9865.21 | 6.82 | 64.80 | 101.91 | -0.352 | nucl: |
| GhPRE13 | Ghi_D02G06716 | 93 | 10648.00 | 9.03 | 88.69 | 94.30 | -0.616 | nucl: |
| GhPRE14 | Ghi_D03G05676 | 91 | 10359.66 | 9.03 | 81.61 | 94.29 | -0.600 | nucl: |
| GhPRE15 | Ghi_D04G02451 | 91 | 10281.70 | 7.98 | 71.87 | 105.16 | -0.360 | mito: |
| GhPRE16 | Ghi_D07G12231 | 94 | 10483.93 | 7.95 | 72.69 | 105.85 | -0.291 | mito: |
| GhPRE17 | Ghi_D09G01021 | 93 | 10568.67 | 9.57 | 87.27 | 73.44 | -1.146 | nucl: |
| GhPRE18 | Ghi_D09G01031 | 92 | 10412.85 | 7.94 | 81.36 | 108.15 | -0.371 | mito: |
| GhPRE19 | Ghi_D11G01701 | 92 | 10419.67 | 7.98 | 68.43 | 95.43 | -0.614 | mito: |
| GhPRE20 | Ghi_D11G01706 | 89 | 9977.29 | 5.20 | 71.11 | 105.17 | -0.328 | nucl: |
| GhPRE21 | Ghi_D12G10716 | 92 | 10354.73 | 9.61 | 74.34 | 98.59 | -0.546 | nucl: |
| GhPRE22 | Ghi_D12G11236 | 87 | 9678.90 | 5.56 | 53.36 | 96.44 | -0.444 | nucl: |
| GhPRE23 | Ghi_D13G03911 | 89 | 9895.17 | 6.25 | 61.90 | 101.91 | -0.344 | nucl: |
| GrPRE1 | Grai_02G012380.1 | 91 | 10359.66 | 9.03 | 81.61 | 94.29 | -0.600 | nucl: |
| GrPRE2 | Grai_03G015460.1 | 93 | 10648.00 | 9.03 | 88.69 | 94.30 | -0.616 | nucl: |
| GrPRE3 | Grai_04G018790.1 | 91 | 10294.70 | 7.98 | 68.28 | 105.16 | -0.391 | mito: |
| GrPRE4 | Grai_05G033250.1 | 93 | 10570.07 | 9.99 | 71.86 | 107.96 | -0.431 | nucl: |
| GrPRE5 | Grai_07G002180.1 | 94 | 10485.90 | 7.95 | 73.60 | 102.77 | -0.344 | mito: |
| GrPRE6 | Grai_09G002410.1 | 101 | 11442.55 | 9.79 | 91.75 | 67.62 | -1.242 | nucl: |
| GrPRE7 | Grai_09G002440.1 | 92 | 10412.85 | 7.94 | 81.36 | 108.15 | -0.371 | mito: |
| GrPRE8 | Grai_11G036210.1 | 89 | 9990.29 | 5.20 | 71.11 | 105.17 | -0.360 | nucl: |
| GrPRE9 | Grai_11G036220.1 | 92 | 10419.67 | 7.98 | 68.43 | 95.43 | -0.614 | mito: |
| GrPRE10 | Grai_12G020930.1 | 92 | 10354.73 | 9.61 | 74.34 | 98.59 | -0.546 | nucl: |
| GrPRE11 | Grai_12G022030.1 | 87 | 9678.90 | 5.56 | 53.36 | 96.44 | -0.444 | nucl: |
| GrPRE12 | Grai_13G008850.1 | 89 | 9887.25 | 6.05 | 60.45 | 106.29 | -0.228 | nucl: |
